# Comparing the metabolomic landscape of polycystic ovary syndrome within urban and rural environments

**DOI:** 10.1038/s43856-025-00985-6

**Published:** 2025-07-01

**Authors:** Jalpa Patel, Hiral Chaudhary, Abhishek Chudasama, Jaydeep Panchal, Akanksha Trivedi, Sonal Panchal, Trupti Joshi, Rushikesh Joshi

**Affiliations:** 1 Department of Biochemistry and Forensic Science, University School of Sciences, Gujarat University, Ahmedabad, Gujarat India; 2 Advait Theragnostics Pvt Ltd, Ahmedabad, Gujarat India; 3 Dr. Nagori’s Institute for Infertility and IVF, Ahmedabad, Gujarat India; 4 Urmi Hospital, Anand, Gujarat India

**Keywords:** Metabolomics, Endocrine reproductive disorders

## Abstract

**Background:**

Polycystic Ovary Syndrome (PCOS) affects up to 10% of women of reproductive age, characterized by hormonal imbalances and metabolic complications. Environmental factors potentially influence the biochemical expression of this condition. This study aims to examine the impact of urban versus rural environments on metabolite profiles in women with PCOS.

**Methods:**

Thirty women aged between 18 and 40, diagnosed with PCOS according to the Rotterdam 2003 criteria, were recruited from June 2022 to May 2023, 16 from urban settings and 14 from rural settings. Serum samples were analyzed using liquid chromatography-tandem mass spectrometry. Principal component analysis and orthogonal partial least squares discriminant analysis were performed to identify metabolic patterns and differences between the two groups.

**Results:**

This study reveals significant differences in metabolite profiles between women with PCOS from various environmental backgrounds. Rural participants exhibit higher levels of lipid-related metabolites, especially Palmitone, indicating specific dietary influences. Urban participants show distinct changes in carbohydrate and nucleotide metabolism pathways, likely due to processed food consumption. Multivariate analyses demonstrate a clear separation between the groups, emphasizing the environmental impact on PCOS expression.

**Conclusions:**

This research highlights potential environment-related biomarkers for PCOS, emphasizing the importance of developing tailored treatment strategies considering environmental factors. The distinct metabolic profiles observed between urban and rural women provide new insights into the syndrome’s complex mechanisms, indicating that environmental influences play a critical role in its biochemical expression and may affect its clinical manifestations.

## Plain language summary

Polycystic Ovary Syndrome (PCOS) is a common condition affecting women’s hormones and metabolism. This study examines how living in a city or rural area affects PCOS. Researchers studied 30 women with PCOS from urban and rural India. They found that rural women had higher levels of certain fats, while urban women had changes in sugar and energy metabolism, likely due to diet. These differences were confirmed using advanced data analysis. The findings show that environment and lifestyle may impact PCOS and its symptoms. Understanding these differences can help doctors create better more personalized treatments for women with PCOS based on where they live and what they eat.

## Abstract (web summary)

Patel et al. explore serum metabolomic differences in PCOS women from urban and rural India. Rural women show higher lipid-related metabolites like Palmitone, while urban women exhibit altered carbohydrate and nucleotide metabolism, likely due to processed food consumption. These findings highlight the impact of lifestyle on PCOS metabolism.

## Introduction

Polycystic Ovary Syndrome (PCOS) is a complex endocrine disorder that stands as a common yet enigmatic challenge in women’s health, characterized by a combination of symptoms such as oligo-anovulation, hyperandrogenism, and polycystic ovarian morphology^1^. Its prevalence underscores not only a significant health burden affecting women of reproductive age worldwide but also a puzzle in medical research due to its heterogeneous nature and multifactorial etiology^2,3^. Emerging evidence suggests that beyond genetic predisposition, environmental factors and lifestyle choices play pivotal roles in the manifestation and severity of PCOS, thus opening new avenues for exploring modifiable risk factors and therapeutic strategies^4–6^.

The metabolome includes all metabolites produced by an organism’s metabolism, and metabolomics provides a snapshot of the physiological state influenced by genetics, environment, and lifestyle^7^. In the context of PCOS, metabolomic analyses offer invaluable insights into the biochemical pathways that are altered by the disorder, potentially unveiling novel biomarkers for early detection, insights into pathogenesis, and targets for treatment^2,8^. Despite the growing body of research on PCOS metabolomics, the differential impact of urban versus rural living environments on the metabolic phenotype of PCOS women remains poorly understood^9^.

The distinction between urban and rural lifestyles encompasses a broad spectrum of factors, including but not limited to dietary habits, physical activity levels, stress, exposure to environmental pollutants, and access to healthcare services^10^. Urbanization is often associated with increased stress, higher exposure to pollutants, and sedentary lifestyles—factors known to exacerbate the risk of metabolic diseases^11^. Conversely, rural environments might offer a different dietary composition and a distinct set of environmental exposures that could influence the metabolomic profile in various ways^12,13^. Understanding how these environmental and lifestyle variables impact the metabolic underpinnings of PCOS is critical for tailoring more effective, personalized intervention strategies.

This manuscript presents a comparative analysis of the serum metabolomic profiles of women with PCOS from urban and rural areas, utilizing the LC-MS/MS technique. The objective is to pinpoint specific metabolites and metabolic pathways influenced by environmental and lifestyle factors. Our results indicate noteworthy differences in metabolite profiles between urban and rural participants. Women in rural areas display higher lipid-related metabolites, especially Palmitone, indicating dietary habits rich in natural fats. Conversely, urban participants show marked changes in carbohydrate and nucleotide metabolism, likely linked to the intake of processed foods. This research deepens our understanding of how environmental contexts affect the metabolic profile of PCOS and aids in the creation of more tailored treatment strategies.

## Methods

### Recruitment of participants and ethical compliance

In this study, we engaged a cohort of 30 women aged between 18 and 40 years, identified with PCOS according to the consensus criteria established by the European Society of Human Reproduction and Embryology (ESHRE) and the American Society for Reproductive Medicine (ASRM), also known as the Rotterdam Criteria. Eligibility for inclusion required participants to manifest at least two of the following: clinical or biochemical signs of androgen excess, recurrent anovulatory cycles, or polycystic ovarian morphology as evidenced by ultrasound.

Participants were categorized based on their environmental background: 16 individuals from urban areas were recruited via Dr. Nagori’s Institute for Infertility and IVF located in Ahmedabad, whereas 14 participants from rural locales were selected from Urmi Hospital in Umreth (Anand). The study protocol received ethical clearance from the Ethical Committee at Gujarat University (reference GU-IEC(NIV)/02/Ph.D./007), adhering to stringent ethical standards and guidelines.

This investigation aimed to dissect the serum metabolomic variations in women diagnosed with PCOS across urban and rural settings through an LC-MS analytical approach. A critical element of the current study design was the application of the Rotterdam 2003 criteria for PCOS diagnosis, which ensured a uniform study population and minimized confounding variables that could influence the study’s outcomes.

In alignment with ethical research practices, informed consent was duly obtained from all participants in both Gujarati and English, ensuring a comprehensive understanding of the study’s objectives among women from varied geographical backgrounds.

### Eligibility criteria

The inclusion criteria for the study require participants to have a confirmed diagnosis of PCOS based on the Rotterdam 2003 guidelines, which necessitate the presence of at least two out of the following three features: oligo- or anovulation, clinical and/or biochemical signs of hyperandrogenism, and polycystic ovarian morphology as observed on ultrasound. Eligible participants must be between 18 and 45, reflecting the age group most commonly affected by PCOS. Additionally, participants must reside in specified urban or rural areas to ensure the study captures the environmental and lifestyle diversity associated with different geographical settings.

The exclusion criteria for the study are designed to eliminate potential confounding factors that could influence the outcomes. Participants with fertility complications not attributable to PCOS are excluded to ensure that the findings are specific to PCOS-related issues. Individuals with known reproductive pathologies, such as tubal blockages or pelvic adhesions, are also excluded, as these conditions may independently affect fertility outcomes. To maintain the integrity of the hormonal profile under investigation, participants who have received hormonal therapies or related medications within six months prior to the study are not eligible. Additionally, individuals who smoke are excluded due to the well-documented effects of smoking on hormonal balance and metabolic health. Finally, those diagnosed with diabetes mellitus are excluded, given the condition’s significant impact on metabolic processes that could confound the study’s results.

It is noteworthy to mention that the urban cohort included participants from an IVF clinic, specifically chosen for their diagnosis of PCOS and not for fertility treatment purposes. This selection aimed at harnessing a broad demographic of PCOS-diagnosed women rather than focusing on their fertility status. Our rigorous exclusion criteria were applied to ensure that any participant with known fertility issues not directly related to PCOS was excluded, thus preserving the integrity and comparability of urban and rural cohorts.

To ensure a balanced representation of women affected by PCOS from both urban and rural areas while minimizing selection bias, participants were recruited based on a confirmed PCOS diagnosis, regardless of their fertility status. The selection of study sites aimed to capture environmental diversity while keeping diagnostic criteria consistent. Strict inclusion and exclusion criteria were uniformly enforced across both groups, ensuring their comparability. Although hospital-based recruitment may not fully represent the broader PCOS population, it facilitated standardized clinical assessments and reduced confounding factors.

### Sample collection and biochemical evaluation

Blood specimens were collected from the participants during the follicular phase (days 2–5) of their menstrual cycle following an overnight fast. This timing was strategic, aiming to optimize the conditions for biochemical assessments and serum metabolomic analysis. The serum samples for this study were collected from January 2022 to October 2022, ensuring a representative capture of metabolic profiles from both urban and rural participants during this period, while also allowing participants to understand the aim of the study. Post-collection, the specimens were centrifuged at 2500 rpm for 15 min, and the serum was subsequently stored at −80 °C until analysis. The biochemical investigations encompassed measurements of luteinizing hormone (LH), follicle-stimulating hormone (FSH), and 17β-estradiol, alongside thyroid-stimulating hormone (TSH) and prolactin (PRL) to preclude thyroid and prolactin disorders. Furthermore, we measured testosterone (T) and dehydroepiandrosterone sulfate (DHEA-S) levels to assess hyperandrogenemia, employing chemiluminescence for all hormonal assays in an accredited clinical laboratory.

### Chemicals

All chemicals were of analytical reagent grade. Ammonium acetate was purchased from Sigma company. Acetonitrile, Water, and Formic acid of LC-MS grade were obtained from J.T Baker.

### Sample preparation for serum metabolomic analysis

Sample preparation for serum metabolomic analysis begins with precisely measuring 100 μl of the serum sample, which is transferred into a microcentrifuge tube. 400 μl of chilled acetonitrile is added to precipitate proteins and facilitate metabolite extraction. The mixture is then vortexed vigorously to ensure optimal integration of the serum and acetonitrile, which is crucial for efficient metabolite extraction. Following vortexing, the sample is incubated at −20 °C for 15 min to enhance further protein precipitation, which aids in their subsequent separation from the metabolites. The mixture is then centrifuged at 12,000 rpm and a temperature of 4 °C for 10 min, a process that allows for the clear separation of the supernatant, which contains the extracted metabolites, from the pellet. Care is taken to transfer the clear supernatant into a new microcentrifuge tube without disturbing the pellet, and the supernatant is then vacuum-dried to remove the acetonitrile, leaving behind the concentrated metabolites. These are resuspended in 300 μl of acetonitrile and filtered through a 0.22 μm nylon syringe filter to eliminate any remaining particulate matter, ensuring the purity of the sample introduced into the LC-MS system.

### Quality control and method validation

A quality control (QC) sample was prepared to evaluate system stability and method performance by mixing equal volumes (10 μL each) of serum from all study samples. This QC sample is analyzed after every sequence of five serum samples to assess system stability and ensure the generation of reliable data throughout the analysis. This approach helps mitigate potential false-positive signals inherent in untargeted metabolomics, especially when analyzing complex samples on the LC-MS platform. Although untargeted analysis offers broad coverage of metabolites, it may lack absolute quantitative data and generate false positives due to the absence of reference standards. Targeted analysis of specific metabolites of interest could address these limitations and provide more accurate qualitative and quantitative insights into the differences between the two groups.

This meticulous preparation and validation process ensures the accuracy, reproducibility, and reliability of serum metabolomic analysis, providing a robust foundation for subsequent LC-MS analysis and a comprehensive exploration of the metabolomic landscape.

### Instrumentation

The instrumental setup for the analysis consisted of a sophisticated Bruker Elute UHPLC system, seamlessly operated through the Hystar 5.0 SR1 software, and was adeptly paired with a Bruker Daltonics AmaZon Speed™ ion trap mass spectrometer, utilizing Data Analysis 5.2 for data processing, all provided by Bruker Daltonics GmbH. Chromatographic separation of the metabolites was proficiently achieved using a Waters UPLC ACQUITY BEH C18 column, featuring 1.7 μm silica particles and dimensions of 50 × 2.1 mm, supplemented with a compatible column guard to ensure consistent analysis quality. The system utilized distinct mobile phase compositions for analyses in both positive and negative ionization modes to optimize metabolite separation. Specifically, the mobile phase for the positive mode consisted of Water with 0.1% formic acid (A) and acetonitrile with 0.1% formic acid (B), whereas for the negative mode, the composition was adjusted to Water with 0.1% ammonium acetate (A) and acetonitrile with 0.1% ammonium acetate (B). Sample extracts were maintained at a thermostated condition of 30 °C within the autosampler. A precise volume of 5 μL of each extract was subjected to chromatographic separation following a meticulous gradient program, starting with 5% of solvent B, which was then progressively increased to 15% at 5 min, 30% at 10 min, peaking at 100% between 16 and 18 min, and then returning to initial conditions by 32 min, with the solvent flow rate set at 300 μL/min. This gradient was designed to ensure comprehensive separation across the total runtime of 32 min for both polarity runs. Column temperature was consistently maintained at 30 °C. Mass spectrometric detection in both positive and negative modes was executed using Auto MS(n) mode with the parameters set as follows: m/z range of 50–1500, capillary voltage at 4.5 kV, nebulizer pressure at 29.0 psi, dry gas flow rate at 10.0 L/min, and a drying gas temperature of 126.9 °C, enabling precise and reproducible metabolomic profiling.

### Metabolomic data analysis

The analysis of MSMS spectra was meticulously conducted by matching against esteemed MSMS libraries, including the Human Metabolome Database (HMDB), Metabolomics Workbench, and the Mass Bank of North America (MoNA), ensuring unparalleled accuracy in metabolite identification. The sophisticated data processing and analysis were carried out using the advanced capabilities of MetaboAnalyst 6.0, a premier online platform dedicated to metabolomics research. The data first underwent a series of preparatory steps to facilitate a robust analytical framework: a logarithmic transformation to stabilize variance, autoscaling to bring all variables to a comparable scale, and sum normalization to correct for sample-to-sample variations. This refined dataset was then analyzed using Principal Component Analysis (PCA) in two and three dimensions, providing a clear visual distinction between the metabolic signatures of Urban-PCOS and Rural-PCOS groups.

Building on this unsupervised approach, a more targeted analysis was conducted through Orthogonal Partial Least Squares Discriminant Analysis (OPLS-DA), enabling a deeper exploration of the metabolic disparities between the two study groups. The quality of the OPLS-DA models was assessed by the goodness of fit (R^2^Y) and the predictive ability of the models (Q^2^). VIP plots were generated to recognize metabolites most significantly responsible for groups separation. Metabolites with VIP value higher than 1.0 were considered potential biomarker candidates. To test the accuracy of the multivariate statistical models and to rule out that the observed separation in the OPLS-DA is due to chance (*p* < 0.05), permutation tests were performed with 2000-fold repetition. The statistical robustness of the metabolite differences observed was rigorously evaluated using paired parametric t-tests, with the Mann–Whitney test and Bonferroni correction applied to address the challenges of multiple testing. Only findings with *P* values and false discovery rates (FDR; *q*-value) below the threshold of 0.05 were considered statistically significant, ensuring the integrity and reliability of the study’s conclusions. Through this comprehensive and meticulous analytical approach, the study adeptly elucidated the nuanced metabolic variations between urban and rural women with PCOS, grounding its findings in rigorous statistical evidence.

### Statistics and reproducibility

This pilot study involved 30 participants to evaluate the hypothesis regarding serum metabolomic differences between urban and rural women with PCOS. The sample size was chosen for feasibility and exploratory purposes, primarily aiming to identify possible metabolic variations that could shed light on future, larger studies.

Each participant served as an independent biological replicate. Blood samples were collected and processed per a standardized protocol to reduce variability. Quality control samples were prepared by pooling serum aliquots from all participants, which were analyzed at regular intervals (after every five study samples) to ensure consistent system performance during LC-MS analysis.

Statistical analysis included data normalization, scaling, and transformations before performing multivariate analyses such as PCA and OPLS-DA. The OPLS-DA models underwent validation through permutation testing with 2000 repetitions to gauge their statistical significance (*p* < 0.05) and robustness. VIP scores exceeding 1.0 indicated metabolites significantly contributing to group differentiation. Paired parametric t-tests were used for univariate comparisons. All analyses were conducted using suitable online platforms (MetaboAnalyst 6.0), and a *p*-value < 0.05 was regarded as statistically significant.

### Reporting summary

Further information on research design is available in the Nature Portfolio Reporting Summary linked to this article.

## Results

### Biochemical and clinical profiles of participants

The present study conducted an untargeted metabolomics analysis via LC-MS/MS on serum samples from PCOS-affected women residing in urban (*n* = 16) and rural (*n* = 14) settings. This analysis aimed to uncover the influence of distinct lifestyle factors, environmental exposures, and dietary habits associated with these geographies on the metabolomic signatures of PCOS. Careful selection ensured that the age and BMI of participants were comparable, eliminating demographic variables as confounding factors (Table 1). The findings of the present study revealed a noteworthy divergence in Luteinizing Hormone (LH) levels, with the rural participants exhibiting significantly higher values. This contrast stood out against a backdrop of uniformity in other measured biochemical markers, including testosterone, estradiol, and thyroid-stimulating hormone (TSH), across both groups (Table 1).

**Table 1:** Demographic and biochemical parameters of PCOS women from urban and rural regions

Demographic variables and biochemical parameters	Urban PCOS (*n* = 16)	Rural PCOS (*n* = 14)	*P*-value
Age (year)	29.25 ± 4.21	27.35 ± 4.41	0.24
Body mass index (BMI) (kg/m^2^)	27.66 ± 5.49	25.86 ± 6.29	0.41
Testosterone (ng/dl)	23.32 ± 12.58	23.84 ± 17.98	0.92
Estradiol (E2) (pg/ml)	65.21 ± 40.35	107.14 ± 73.39	0.058
Dehydroepiandrosterone sulfate (DHEAS) (ug/dl)	120.18 ± 34.54	94.26 ± 38.89	0.063
Thyroid-stimulating hormone (TSH) (uIU/ml)	2.06 ± 1.20	1.44 ± 0.83	0.12
Luteinizing hormone (LH) (mIU/ml)	6.49 ± 3.41	10.22 ± 5.82	0.038
Follicle-stimulating hormone (FSH) (mIU/ml)	8.87 ± 3.82	8.52 ± 3.08	0.78
Prolactin (ng/ml)	13.35 ± 4.11	22.69 ± 26.03	0.16
Luteinizing hormone/Follicle-stimulating hormone (LH/FSH)ratio	0.85 ± 0.56	1.26 ± 0.82	0.12

All data presented in the table is in mean ± SD.

### General characteristics of serum metabolites in the two groups

**Fig. 1:**
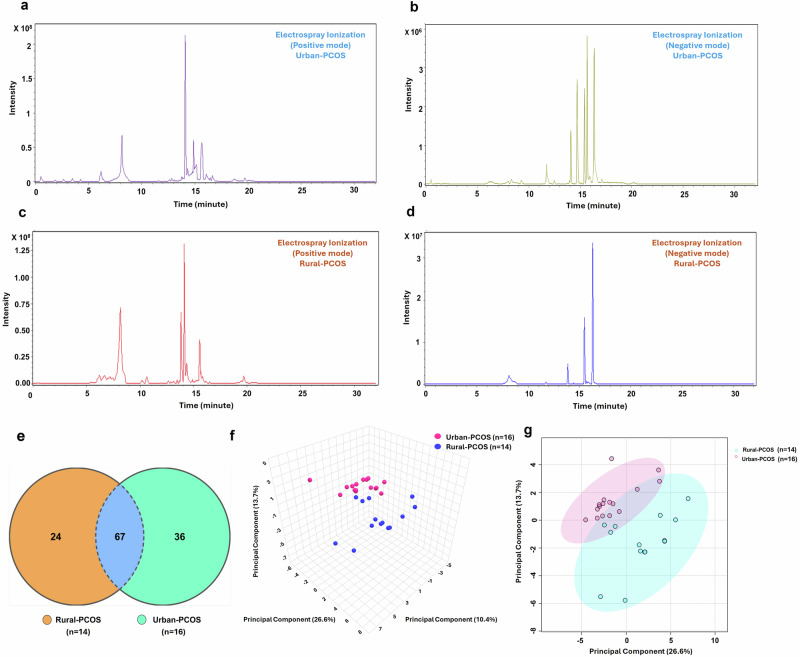
Analytical characterization and comparative metabolomic profiling in urban-PCOS and rural-PCOS. Initial chromatograms (panels **a**–**d**) illustrate distinct peak intensities across ionization modes, the Venn diagram (panel **e**) enumerates metabolites contributing to the study, and PCA (panels **f**, **g**) elucidates the distinct metabolic clustering between the cohorts.

In the analytical phase of the study, as shown in Fig. 1, untargeted serum metabolomics was conducted to reveal the differences in metabolic profiles between the Urban-PCOS and Rural-PCOS groups. The chromatograms in panel a–d (Fig. 1) demonstrate the analytical performance of the selected methodology, displaying the peak intensities of metabolites under both positive and negative ionization modes. These foundational chromatographic results offer a clear visual differentiation between the two cohorts, with distinct chromatographic fingerprints emerging for each group.

The metabolite identification process was further refined through comparative analysis, leading to the construction of a Venn diagram in panel e (Fig. 1). This diagram was instrumental in visualizing the overlap and uniqueness of metabolites identified across the groups based on cross-referencing with multiple metabolite databases. We have selected 37 relevant metabolites from the total pool identified for in-depth analysis.

To capture the variance and relationships within the metabolomic data, we conducted principal component analysis (PCA) and presented the results in panels f and g (Fig. 1). The PCA plots visually depict the distribution of samples along the principal components, confirming the distinction between the Urban-PCOS and Rural-PCOS groups. This multivariate analysis validates the group differentiation observed in the chromatograms and emphasizes the metabolic disparities that are potentially influenced by urban and rural environments.

### Differential metabolite expression in PCOS across rural and urban populations

**Fig. 2:**
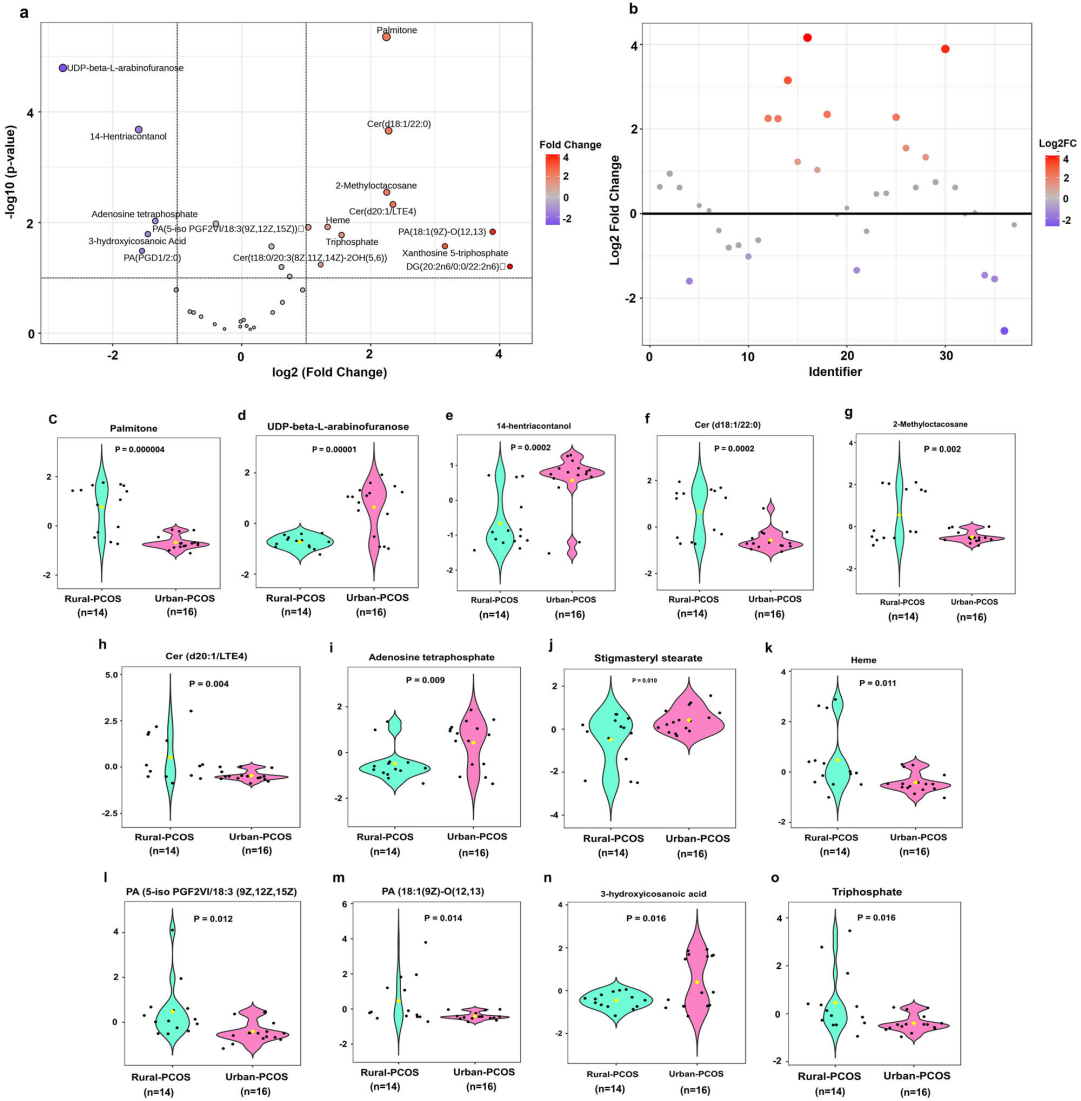
Metabolomic analysis reveals distinct metabolite profiles between rural and urban PCOS patients. **a** Volcano plot visualizing significantly altered metabolites, with log2 fold-change (FC) on the x-axis and -log10 *p*-value on the y-axis, demarcating the threshold of significance. Metabolites to the right are predominantly upregulated in Urban-PCOS, and those to the left are upregulated in Rural-PCOS. **b** Fold-change plot categorizing metabolites by the magnitude of their expression differences between the groups. **c**–**o** Violin plots showing the distribution of selected significant metabolites across the two cohorts, with statistical annotations (*p*-values) indicating the significance level. Statistical comparisons were performed using an unpaired two-tailed t-test. These plots elucidate the specific metabolite alterations within the comparative context of rural versus urban living environments in PCOS patients.

The metabolomic profiling study has revealed distinct variations in metabolite expression between PCOS patients living in rural and urban settings (Supplementary Table 1). Illustrated in data visualization (Fig. 2a, Supplementary Table 2), the volcano plot highlights significant metabolites; those in the upper left are denoted by orange dots indicating downregulation, and those in the upper right by purple dots indicating upregulation.

Among the metabolites, Palmitone is notably upregulated in Rural-PCOS, with a log_2_ fold change (FC) of 2.2447, signifying increased lipid accumulation potentially linked to rural lifestyle factors. In stark contrast, UDP-beta-L-arabino furanose is significantly downregulated in Rural-PCOS, with a log_2_ FC of −2.7718, suggesting it is more prevalent in Urban-PCOS and possibly indicative of distinct urban carbohydrate metabolism pathways.

Lipid-related metabolites such as Cer(d18:1/22:0) and 2-Methyloctacosane also demonstrate marked upregulation in Rural-PCOS, with log_2_ FCs of 2.2781 and 2.2495, respectively. This trend continues with Cer(d20:1/LTE4) and DG (20:2n6/0:0/22:2n6), which exhibit significant upregulations with log_2_ FCs of 2.3458 and 4.1598, pointing to a pronounced alteration in lipid signaling and storage specific to the rural environment.

Conversely, 3-hydroxyicosanoic Acid and PA(PGD1/2:0) show reductions in Rural-PCOS, with log_2_ FCs of −1.4556 and −1.5458, respectively, indicating a decrease in certain lipid and phospholipid metabolites relative to their urban counterparts. Further insights are provided by the upregulation of metabolites like Heme and Triphosphate in Rural-PCOS, with log_2_ FCs of 1.3325 and 1.5484, respectively, suggesting adaptations in oxidative stress response and energy metabolism that may be more pronounced in rural settings. Additionally, Xanthosine 5-triphosphate is significantly upregulated, with a log_2_ FC of 3.1518, highlighting substantial shifts in nucleotide metabolism specific to Rural-PCOS.

In the context of the fold-change plot (Fig. 2b, Supplementary Table 3), several metabolites showed notable differences in the Urban-PCOS group, demonstrating a shift in metabolic pathways that could be related to urban lifestyle factors. This plot serves to pinpoint the extent of upregulation or downregulation of these metabolites, with the color gradient representing the logarithmic scale of fold-change, reinforcing the magnitude of their differential expression.

The series of violin plots comprise t-test analysis (Fig. 2c–o, Supplementary Table 4) further detail the individual metabolite levels. Metabolites such as palmitoleic acid (Fig. 2c) and UDP-beta-L-arabino furanose (Fig. 2d) were significantly elevated in the urban cohort. This contrasts with heme (Fig. 2k), which demonstrated a notable increase in the Rural-PCOS group. The spread and median of the data points within these plots illustrate the distribution pattern across both cohorts, offering a window into the variations in metabolic expression.

**Fig. 3:**
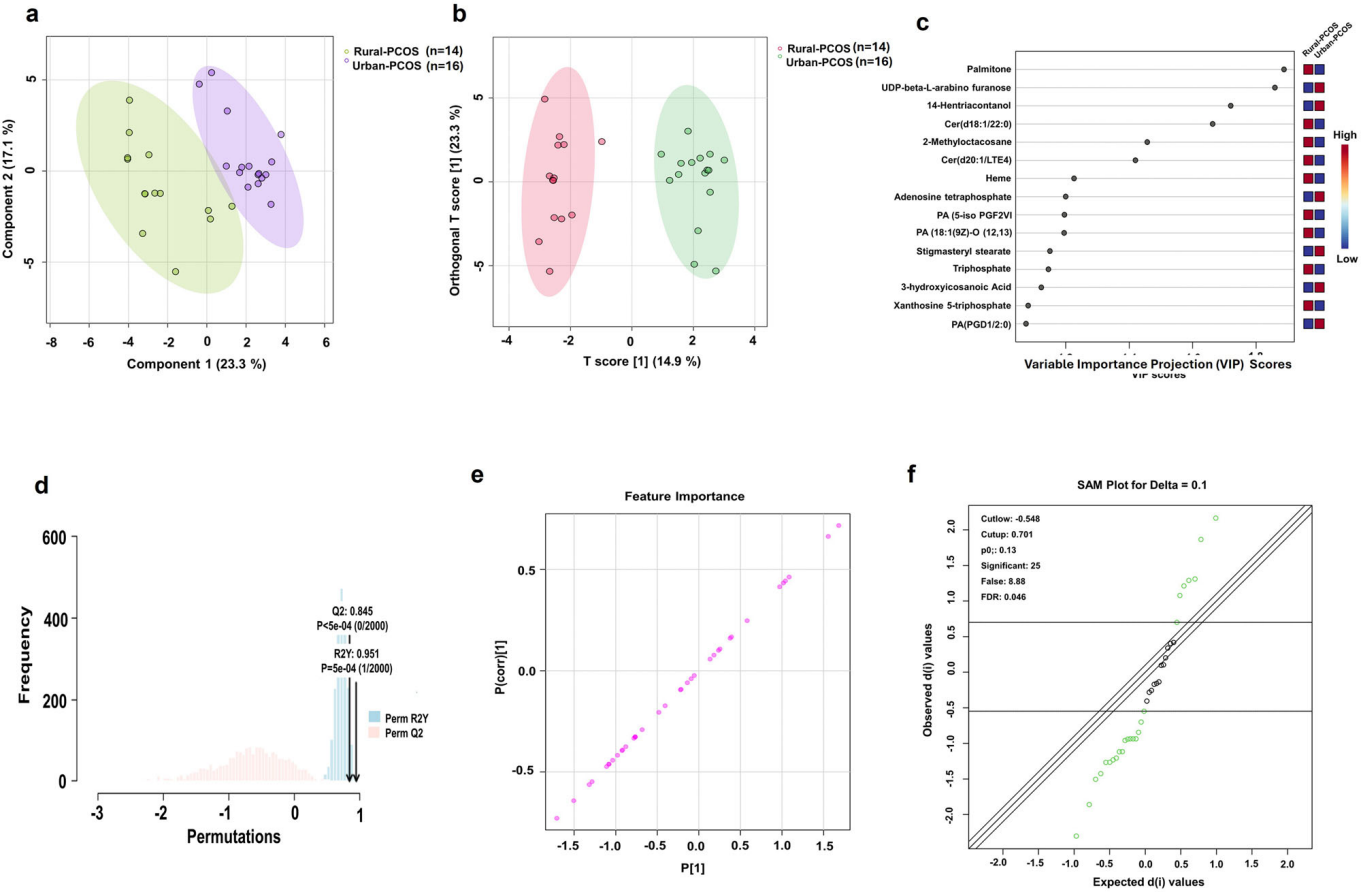
Metabolomic differentiation of PCOS in rural versus urban environments. **a** PLS-DA scatter plot distinguishing Rural-PCOS (green) and Urban-PCOS (purple) groups along two principal components representing the most significant variance within the dataset. **b** OPLS-DA score plot enhances the metabolite-based separation between the groups, with T-scores depicting the discriminatory power. **c** VIP scores from the PLS-DA model rank the metabolites by their contribution to the group differentiation, with higher scores indicating greater importance. **d** The permutation test validated the PLS-DA model, with observed R2 and Q2 values far exceeding those from randomly permuted data, underscoring model reliability. **e** The feature importance plot displays the magnitude and reliability of each metabolite’s contribution to the model. **f** SAM plots for significant differential expression of metabolites between groups at a specified delta, demonstrating robust statistical significance with a low false discovery rate.

Our multivariate statistical analysis illustrates a discernible separation in metabolomic profiles between rural PCOS and urban PCOS patients. In Fig. 3a, the PLS-DA plot demonstrates clear segregation of the two cohorts, with principal components 1 and 2 capturing 23.3% and 17.1% of the variance, respectively. This pronounced clustering is indicative of distinct metabolic signatures attributable to each group. The Rural-PCOS cluster is visibly separate from the Urban-PCOS cluster. Likewise, the OPLS-DA score plot, Fig. 3b, supports the initial PCA findings by further resolving the metabolic distinctions into discriminative components that capture 14.9% of the model’s variance. The metabolites contributing to this separation are identified through their T-scores, emphasizing the potential of metabolomic profiling in elucidating the complex pathophysiology of PCOS as it intersects with environmental factors.

In assessing the contributions of metabolites to the model, VIP scores presented in Fig. 3c (Supplementary Table 5) identify Palmitone, UDP-beta-L-arabino furanose, and 14-Hentriacontanone as significant contributors to the variance between the Rural-PCOS and Urban-PCOS groups. These metabolites, which hold VIP scores indicative of their discriminatory power, may serve as focal points for understanding how environmental factors could drive the metabolic alterations seen in PCOS.

Furthermore, the permutation test in Fig. 3d demonstrates the statistical strength of the model, with high R2 and Q2 values indicative of a robust fit and predictive ability. The low *p*-values associated with these metrics confirm the reliability of the observed separation between the Rural-PCOS and Urban-PCOS groups, suggesting that the model is well-tuned to the underlying data structure and not a product of random variation.

Moreover, Fig. 3e offers a feature important plot that ranks the metabolites according to their impact and correlation within the model. The plot illustrates a spectrum of metabolites with varying degrees of importance, providing a measure not only of their contribution to the classification model but also of their relevance to the biological differences between the patient groups. Likewise, Fig. 3f (Supplementary Table 6) depicts the Significance Analysis of Microarrays (SAM) plot, which identifies metabolites whose expression differences are statistically significant beyond expected random variation at the set delta value. With the low false discovery rate (FDR = 0.006) and 25 metabolites flagged as significant, the SAM plot underscores the reliability of these metabolites as robust biomarkers, thereby enriching the interpretive value of the metabolomic analysis.

### Correlational analysis between metabolites and clinical parameters

**Fig. 4:**
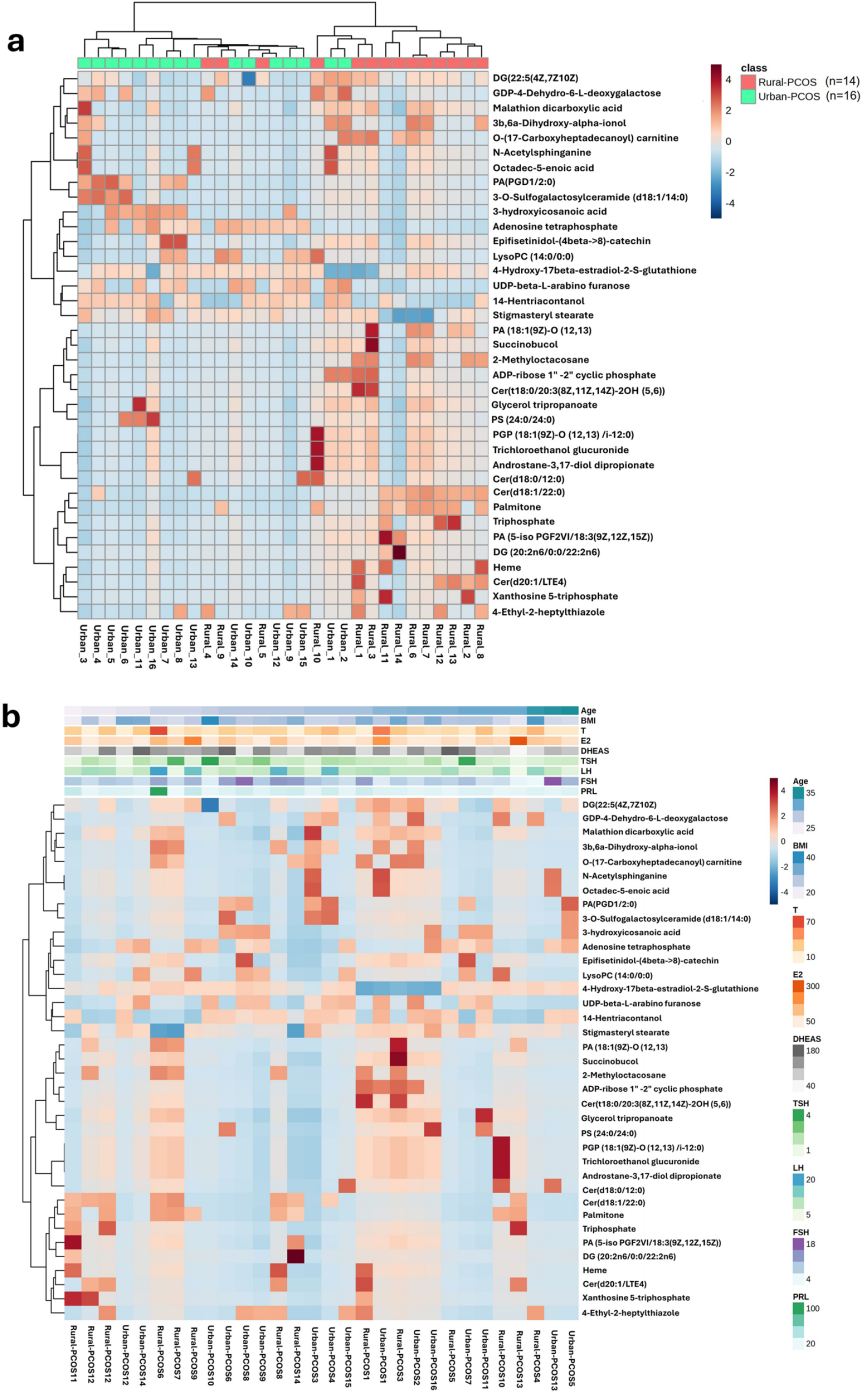
Comparative metabolomic profiling in PCOS of rual and urban subjects. **a** Correlation heatmaps depicting the serum metabolite associations in Rural-PCOS versus Urban-PCOS patients, **b** the Pearson correlation of the relationship between metabolites and biochemical parameters across both cohorts.

The heatmap depicted in Fig. 4 offers a global overview of metabolic features, incorporating identified differential metabolites in two distinct groups, PCOS and urban PCOS, and the correlational analysis of these metabolites with various biochemical parameters. Figure 4a features a correlation matrix for various metabolites, indicated on the y-axis, against samples from participants, indicated on the x-axis, Rural-PCOS and Urban-PCOS. Each cell within the matrix is color-coded, indicating the strength of correlation: red for positive correlation and blue for negative correlation between the metabolite levels in each sample. The dendrogram (tree-like diagram) above the heatmap suggests how the samples cluster together based on their metabolic profiles, with distinct groupings for rural versus urban populations, while the dendrogram to the left indicates clusters among metabolites.

Figure 4b shifts focus to correlate these same metabolites with various physiological and biochemical parameters, such as age, BMI, and hormone levels like testosterone (T), estrogen (E2), and others. The colors in this heatmap correspond to the strength of the relationship between the metabolite concentrations and these parameters. The clustering pattern here provides insight into how certain metabolites may influence, or be influenced by, these physiological and biochemical factors within the context of PCOS.

Notably, the heatmap elucidates a distinct correlation profile for several key metabolites across the two PCOS groups. These differences in metabolite correlation could reflect underlying variations in environmental exposures or lifestyle factors between the rural and urban settings. The strength and pattern of these correlations provide a metabolic fingerprint that could potentially be used to differentiate between rural and urban PCOS phenotypes, offering a new dimension in the understanding of the metabolic underpinnings of PCOS and its environmental context.

**Fig. 5:**
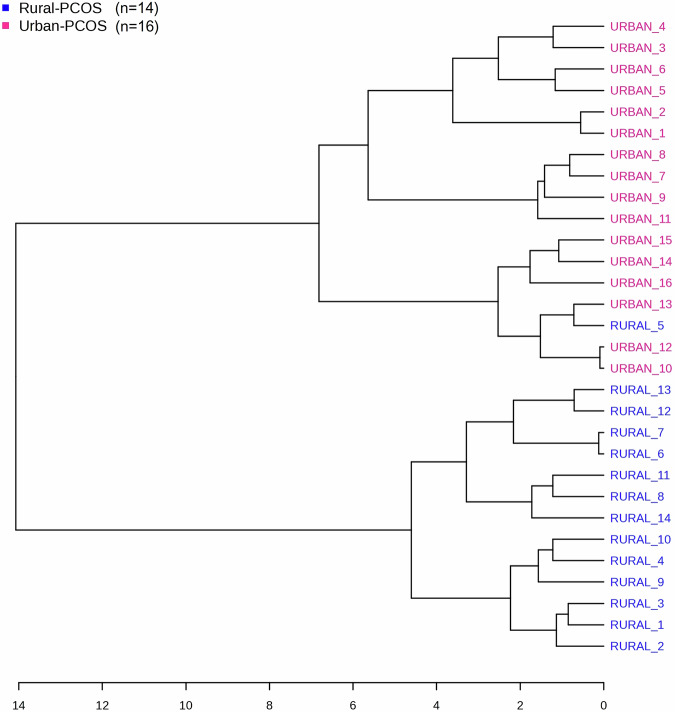
Hierarchical clustering dendrogram of PCOS features in rural and urban subjects using Spearman’s rank correlation. The dendrogram segregates subjects into distinct clusters based on the similarity of their features, with a clear delineation between rural and urban populations.

In Fig. 5, we present a dendrogram derived from hierarchical cluster analysis using Spearman’s rank correlation coefficient as the measure of association. This visual representation delineates the phenotypic clustering of PCOS characteristics among subjects residing in rural and urban environments. The vertical axis of the dendrogram denotes the Spearman distance, with shorter branches correlating to stronger rank correlations between the features of the subjects.

Upon examination, the dendrogram bifurcates primarily into two overarching clusters, which correspond to the rural and urban subsets. Within these predominant clusters, further subdivision is evident, highlighting intra-group similarity and inter-group variability of PCOS features. Notably, ‘Urban-PCOS’ subjects demonstrate a broad spectrum of phenotypic diversity, as indicated by the formation of subclusters at higher Spearman distances. This contrasts with the ‘Rural-PCOS’ cluster, where subjects are linked at notably shorter distances, implying a more homogenous phenotypic expression within this demographic.

The clustering pattern observed suggests a potential influence of environmental factors inherent to rural or urban settings on the expression of PCOS characteristics. These results underscore the utility of non-parametric approaches, such as Spearman’s rank correlation, in elucidating the complex phenotypic landscape of PCOS in varied populations.

### Biomarker analysis of identified metabolites

Comprehensive biomarker analysis of identified metabolites by multivariate analysis highlights the predictive performance of specific biomarkers in discriminating between rural PCOS and urban PCOS patients. Figure 5a presents ROC curves that span a range of models, each incorporating an increasing number of metabolite features. These curves are characterized by their respective AUC values, which impressively peak at 0.983 in the model utilizing 37 metabolites. This peak, situated within a confidence interval that approaches perfection, underscores the exceptional predictive precision of the full metabolite panel.

**Fig. 6:**
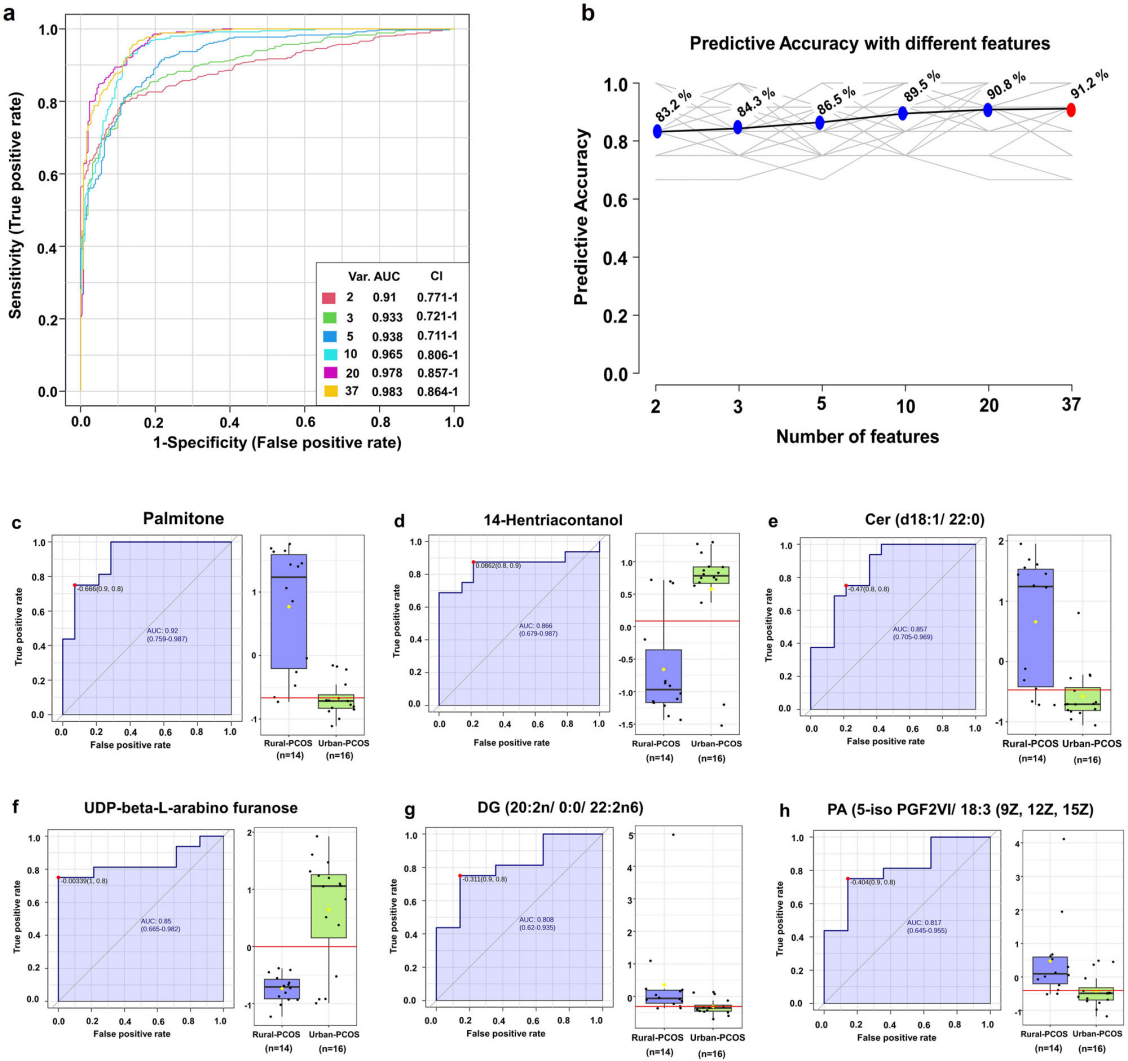
Integrated biomarker analysis of metabolomic profiles in PCOS. **a** ROC curves with AUC values for models incorporating different numbers of metabolites. **b** illustrates the predictive accuracies across models, identifying an optimal number of features for maximal accuracy. **c**–**h** present box plots and ROC curves for selected high-AUC metabolites, emphasizing their role as key biomarkers in differentiating Rural-PCOS from Urban-PCOS phenotypes. Statistical significance in box plots was assessed using an unpaired two-tailed t-test.

In Fig. 6b, the exploration of predictive accuracy reveals a discernible trend: as the number of metabolite features in the model increases, so does the accuracy of classifying PCOS patients into rural or urban categories. This upward trajectory, marked by blue points, suggests an enhanced ability of the model to leverage a more comprehensive metabolite profile for accurate classification. A key observation is the plateau in accuracy gains beyond the inclusion of ten features, highlighted by the red point. This suggests an optimal number of metabolites for maximal classification efficacy, indicating a threshold beyond which additional features do not translate into significant performance benefits.

For a detailed understanding of individual metabolite performances, Supplementary Table 7 lays the groundwork by listing the ROC analysis outcomes for all the examined metabolites. Building on this, Fig. 6c–h offers a focused visualization for those metabolites with the highest biomarker potential. Each panel pairs a ROC curve demonstrating sensitivity and specificity with a box plot detailing the distribution differences in metabolite levels between the Rural and Urban PCOS groups. These visualizations were judiciously selected to highlight metabolites such as Palmitone (Fig. 6c), 14-Hentriacontanol (Fig. 6d), Cer(d18:1/22:0) (Fig. 6e), UDP-beta-L-arabino furanose (Fig. 6f), DG (20:2n6/0:0/22:2n6) (Fig. 6g), and PA (5-iso PGF2VI/18:3 (9Z,12Z,15Z) (Fig. 6h) which not only exhibit high AUC values but also represent the most pronounced differences in expression levels, marking them as key discriminative biomarkers.

### Pathway enrichment analysis of metabolomics

**Fig. 7:**
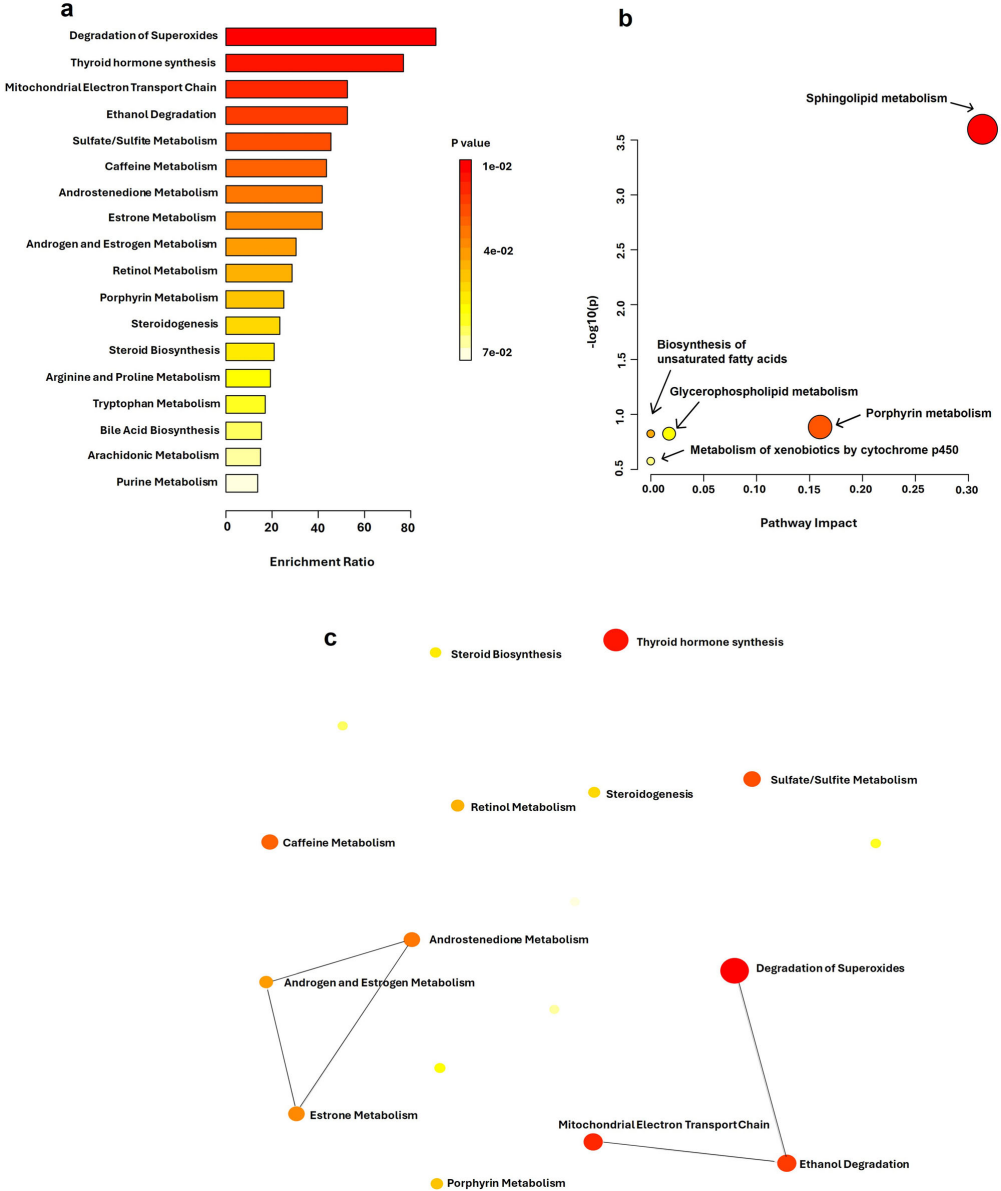
Pathway analysis of the differential metabolites between rural-PCOS versus urban-PCOS. **a** Enrichment analysis of metabolic pathways. The bar chart illustrates the enrichment ratios of significantly altered metabolite sets, with color intensity denoting the statistical significance (*p*-value) for each pathway. Statistical significance was determined using a hypergeometric test, and *p*-values were adjusted using the Benjamini–Hochberg method for multiple testing correction. **b** Pathway enrichment analysis of differential metabolites between PCOS and control groups. Significant metabolites were identified using an unpaired two-tailed t-test with FDR correction (*p* < 0.05). Pathway enrichment was performed using the over-representation method based on the hypergeometric test. **c** Network plot displaying the interconnectedness of metabolically altered pathways in PCOS.

The metabolite enrichment analysis, as depicted in Fig. 7a, provided a quantitative overview of metabolic pathways potentially implicated in the condition under investigation. The analysis revealed a pronounced enrichment of the ‘Degradation of Superoxides’ pathway, denoted by the most substantial bar and a deep red hue, indicating a high enrichment ratio and robust statistical significance (*p*-value < 0.01). Similarly, the ‘Thyroid hormone synthesis’ and ‘Mitochondrial Electron Transport Chain’ pathways exhibited significant enrichment, underscoring their possible roles in the metabolic alterations observed.

Conversely, pathways such as ‘Purine Metabolism’ exhibited minimal enrichment, as evidenced by shorter bars and a color shift towards yellow, suggesting a weaker association with the experimental condition. The color gradient from red to yellow across the bars illustrated a spectrum of statistical significance across the various metabolic pathways, with red indicating higher significance.

Panel b (Fig. 7) could be a scatter plot, often referred to as a volcano plot, where the x-axis represents pathway impact, and the y-axis shows the -log (*p*-value). Each dot represents a metabolic pathway, with the size likely reflecting the magnitude of the pathway’s impact or the number of hits within the pathway. The position of a dot higher on the y-axis suggests greater statistical significance.

Panel c (Fig. 7) appears to be a network plot illustrating the interconnections between different metabolic pathways. The different colors of the dots likely indicate different categories or types of pathways, with lines suggesting relationships or shared components between pathways. The size of each dot might indicate the relative importance or impact of each pathway within the network.

## Discussion

PCOS is a prevalent endocrine disorder that affects ~8–13% of women of reproductive age, characterized by a range of symptoms that can include menstrual irregularities, hyperandrogenism, and polycystic ovaries. The prevalence of disorders and manifestations vary significantly between different geographical locations, influenced by distinct environmental, lifestyle, and dietary factors^14^. In India, the prevalence of PCOS is higher in urban areas, particularly among middle-class women, compared to rural areas^15^. Urban women often encounter lifestyle stressors such as higher stress levels and consumption of processed foods, which are linked to exacerbated symptoms of PCOS^16^. In contrast, women in rural areas might experience variations in diet and physical activity levels, potentially influencing the disease’s manifestation differently^17^. These geographical disparities in the prevalence and impact of PCOS highlight a critical gap in our understanding and management of the disorder, making it a significant public health issue^18^.

Given the complex interplay of genetic, metabolic, and environmental factors in PCOS, the present study utilized untargeted metabolomics to explore the biochemical and metabolic distinctions between urban and rural women diagnosed with the condition. This approach is novel in adding a geographical dimension to the metabolic profiling of PCOS, which has traditionally focused on comparisons between PCOS patients and healthy controls^19,20^. These findings reveal significant metabolic variations that reflect these women’s distinct environments, thereby providing deeper insights into how local lifestyle factors can influence disease pathology.

The significant variation in Luteinizing Hormone (LH) levels between the rural and urban cohorts, with higher levels observed in the rural participants, is a pivotal finding. Elevated LH is a hallmark of PCOS and is known to exacerbate the condition by promoting ovarian androgen production^21^. The divergence observed could be attributed to differences in lifestyle factors such as stress, diet, and physical activity, which are known to influence the hypothalamic-pituitary-gonadal axis. This aligns with J. Patel et al.^9^, who suggests that rural environments might offer different stressors and dietary exposures that affect hormonal regulation differently than urban settings.

The significant segregation of metabolic profiles between the urban and rural PCOS groups, as evidenced by PCA and OPLS-DA results, supports the hypothesis that lifestyle factors intrinsic to these environments contribute uniquely to the expression of disease. For instance, the increase in lipid-related metabolites like Palmitone in rural participants points towards potential differences in diet or metabolic responses to diets richer in saturated fats, which are more common in rural diets^22–24^. This insight is crucial for understanding how localized lifestyle interventions could be tailored to manage PCOS symptoms more effectively.

The identification of unique carbohydrate and nucleotide metabolism pathways in the urban cohort, mainly through metabolites like UDP-beta-L-arabino furanose and Xanthosine 5-triphosphate, underscores the impact of urban dietary habits, which typically include higher consumption of processed foods and sugars. This dietary pattern is especially relevant in the context of managing insulin resistance—a prevalent complication in PCOS—suggesting that urban living may intensify certain metabolic disruptions linked to the disorder^25^. The connection between urbanization, dietary choices, and their role in exacerbating metabolic disturbances such as insulin resistance in PCOS is compelling. This relationship is accentuated by the broader influence of environmental factors, which can significantly affect the clinical manifestation of PCOS, underscoring the critical need for targeted dietary and lifestyle interventions in these settings^26^.

The pathway enrichment analysis has highlighted significant roles for oxidative stress and altered energy metabolism in PCOS, emphasizing the involvement of critical pathways such as ‘Degradation of Superoxide,’ ‘Thyroid hormone synthesis,’ and the ‘Mitochondrial Electron Transport Chain.’ These insights suggest that metabolic dysfunctions in PCOS are not only widespread but also influenced by environmental factors, which can be leveraged to develop targeted therapeutic interventions. This underscores the necessity of a holistic approach in both the diagnosis and treatment of PCOS.

PCOS is intricately linked with oxidative stress and compromised energy metabolism, where mitochondrial dysfunction plays a central role. Such dysfunction may lead to oxidative damage to mitochondrial DNA, exacerbating the metabolic anomalies seen in PCOS^27,28^. Additionally, oxidative stress impacts the phosphoprotein phosphatase 2A (PP2A) system—a pivotal regulator of cellular processes—pointing to possible therapeutic avenues^29^. The interplay between oxidative stress and metabolic processes is crucial for understanding the molecular foundations of PCOS and other age-related diseases^30^.

These findings advocate for a comprehensive and nuanced approach to managing PCOS, emphasizing the importance of addressing oxidative stress and mitochondrial dysfunction. By targeting pathways such as ‘Degradation of Superoxide,’ ‘Thyroid hormone synthesis,’ and the ‘Mitochondrial Electron Transport Chain,’ treatment strategies can be refined to mitigate the underlying metabolic disturbances characteristic of PCOS. This strategic focus on pathophysiological pathways opens new doors for advancing PCOS management and therapy, aligning with a more personalized medicine approach.

The discovery of robust biomarkers like Palmitone and Cer (d18:1/22:0) has significantly enriched our understanding of PCOS, highlighting the relevance of lipid metabolism in its pathophysiology^31,32^. These biomarkers have demonstrated high predictive accuracy in distinguishing PCOS phenotypes in different environments, underscoring the influence of environmental contexts on the disorder^31^. This finding aligns with the broader understanding of PCOS as a complex condition influenced by both genetic and environmental factors^33^.

The distinct metabolic variations observed between urban and rural PCOS patients highlight the necessity of personalized treatment strategies tailored to their specific metabolic and lifestyle-related risk factors. Urban PCOS patients, displaying altered carbohydrate metabolism and increased oxidative stress, may benefit from targeted dietary interventions such as a low-glycemic index diet, antioxidant supplementation, and structured exercise programs like resistance training to improve insulin sensitivity. Conversely, rural patients with significant metabolic disturbances may require omega-3-rich diets, lipid-lowering interventions, and balanced exercise routines. Pharmacological approaches, including metformin or inositol-based therapies, could be considered based on metabolic profiles. These findings emphasize the need for region-specific metabolic screening and risk assessment models to guide early diagnosis and intervention. Follow-up longitudinal studies will be essential in tracking metabolic changes over time, establishing causal links between environmental exposures and disease progression, and refining therapeutic strategies for better clinical management of PCOS.

Our research significantly advances the emerging field of PCOS metabolomics, as few studies have explored metabolic changes in women with PCOS^34,35^. To our knowledge, this is the first study to investigate metabolomic differences between urban and rural PCOS women, offering new insights into how regional factors influence metabolic profiles. This perspective enhances our understanding of the environmental and lifestyle impacts on metabolic dysregulation associated with PCOS. Our findings lay the groundwork for future personalized treatment approaches, as identifying distinct metabolic signatures in different PCOS subpopulations may enable targeted therapeutic interventions.

However, our study does have limitations. One primary concern is the potential influence of lifestyle-related confounding factors, including diet, physical activity, and stress, which can differ significantly between urban and rural groups and may affect metabolomic profiles. While we collected self-reported lifestyle data, our analysis did not quantitatively assess or control for these factors. Future research incorporating comprehensive dietary intake assessments, objective physical activity monitoring, and validated stress evaluation tools will be crucial to accurately understand their role in PCOS-related metabolic changes.

Additionally, our study is limited to one region in India, which may affect the generalizability of our findings to other regions or ethnic groups. Expanding the study to include diverse geographical and ethnic populations will help validate the observed metabolic differences and improve the applicability of our results to a broader PCOS population. Furthermore, while our research identifies unique metabolic signatures within PCOS subpopulations, it does not establish a causal relationship between these metabolic variations and regional lifestyle characteristics. Longitudinal studies with controlled lifestyle interventions and larger sample size will be essential to strengthen statistical power, confirm our findings, and refine personalized treatment strategies for PCOS management.

Conclusively, this study marks a significant advance in the field of PCOS research, being the first to apply untargeted metabolomics to investigate the influence of urban and rural environments on the metabolic profiling of the disorder. It opens new avenues for research into how environmental factors affect the etiology and progression of PCOS and lays the groundwork for future longitudinal studies that will further elucidate these relationships. Such studies are crucial for developing nuanced, location-specific treatment modalities that consider the complex interplay between genetics, metabolism, and environmental factors in PCOS.

## Supplementary information

Description of Additional Supplementary files

Supplementary Data 1

Supplementary Data 2

Supplementary Data 3

Supplementary Data 4

Supplementary Data 5

Supplementary Data 6

Supplementary Data 7

Supplementary Data 8

Reporting Summary

## Data Availability

The mass spectrometry data for untargeted serum metabolomics is accessible at 10.6084/m9.figshare.28891628(https://figshare.com/articles/dataset/_b_Comparing_the_Metabolomic_Landscape_of_Polycystic_Ovary_Syndrome_within_Urban_and_Rural_Environments_b_/28891628)^36^. Supplementary Table 1 shows the Characteristics of differential serum metabolites of Rural and Urban PCOS women. The source data for Fig. 2a is provided in Supplementary Table 2, for Fig. 2b in Supplementary Table 3, and for Fig. 2c–o in Supplementary Table 4. The source data for Fig. 3c is available in Supplementary Table 5, and for Fig. 3f in Supplementary Table 6. Additionally, Supplementary Table 7 contains the Receiver Operating Characteristic (ROC) analysis results for biomarker identification, and Supplementary Table 8 contains the source data of Fig. 7b.

## Abbreviations

PCOS: polycystic ovary syndrome
BMI: body mass index
WHR: waist to hip ratio,
FSH: follicle stimulating hormone
LH: luteinizing hormone
TSH: thyroid stimulating hormone
DHEAS: dehydroepiandrosterone sulfate,
PRL: prolactin
E2: estradiol
T: testosterone
LC-MS: liquid chromatography-mass spectrometry
OPLS-DA: orthogonal partial least squares discriminant analysis
PLS-DA: partial least squares discriminant analysis
PCA: principal component analysis
FDR: false discovery rate
SAM: significance analysis of microarrays
ROC: receiver operating characteristic
VIP: variable importance projection
