# Supplementary material for: Comparing the metabolomic landscape of polycystic ovary syndrome within urban and rural environments
Source: Commun Med (Lond). 2025 Jul 1;5:253. doi: 10.1038/s43856-025-00985-6 (PMC12214864; doi:10.1038/s43856-025-00985-6)
Supplement: Supplementary file 1 — Description of Additional Supplementary files [file 43856_2025_985_MOESM1_ESM.pdf]

Description of Additional Supplementary files

File name: Supplementary Data 1

Description: Characteristics of differential serum metabolites of Rural and Urban PCOS women.

File name: Supplementary Data 2

Description: Volcano plot analysis of differential metabolites.

File name: Supplementary Data 3

Description: Fold change analysis of differential metabolites

File name: Supplementary Data 4

Description: T-test analysis of differential metabolites between Rural PCOS and Urban PCOS groups.

File name: Supplementary Data 5

Description: The rank of the metabolites by their contribution to the group differentiation with scores by Partial Least Squares Discriminant Analysis.

File name: Supplementary Data 6

Description: Significant differential expression of metabolites by Significant analysis of microarrays (SAM)

File name: Supplementary Data 7

Description: Receiver Operating Characteristic (ROC) analysis for Biomarker identification.

File name: Supplementary Data 8

Description: Pathway enrichment analysis of differential metabolites between PCOS and control groups.
