# Supplementary material for: Comparing the metabolomic landscape of polycystic ovary syndrome within urban and rural environments
Source: Commun Med (Lond). 2025 Jul 1;5:253. doi: 10.1038/s43856-025-00985-6 (PMC12214864; doi:10.1038/s43856-025-00985-6)
Supplement: Supplementary file 2 — Supplementary Data 1 [file 43856_2025_985_MOESM2_ESM.docx]

**Comparing the Metabolomic Landscape of Polycystic Ovary Syndrome within Urban and Rural Environments**

Jalpa Patel^1^, Hiral Chaudhary^1^, Abhishek Chudasama^1^, Jaydeep Panchal^2^, Akanksha Trivedi^2^, Sonal Panchal^3^, Trupti Joshi^4^, Rushikesh Joshi^1*^

^1^Department of Biochemistry and Forensic Science, University School of Sciences, Gujarat University, Ahmedabad-380009, Gujarat, India.

^2^Advait Theragnostics Pvt Ltd, Ahmedabad- 380009, Gujarat, India.

^3^Dr. Nagori's Institute for Infertility and IVF, Ahmedabad-380009, Gujarat, India.

^4^Urmi Hospital, Umreth-388220, Anand, Gujarat, India.

***Correspondence:**

Dr. Rushikesh Joshi, ​

Assistant Professor,

Department of Biochemistry & Forensic Science,

University School of Sciences,

Gujarat University, Ahmedabad-380009, India.

Email ID: [rushikeshjoshi@gujaratuniversity.ac.in](mailto:rushikeshjoshi@gujaratuniversity.ac.in)

**Author’s information**

Jalpa Patel: [jalpa.patel515@gmail.com](mailto:jalpa.patel515@gmail.com)

Hiral Chaudhary: [hiralchaudhary54@gmail.com](mailto:hiralchaudhary54@gmail.com)

Akanksha Trivedi: [akanksha.m1323@gmail.com](mailto:akanksha.m1323@gmail.com)

Abhishek Chudasama: [abhichudasama@gmail.com](mailto:abhichudasama@gmail.com)

Jaydeep Panchal: panchaljaydeep80@gmail.com

Sonal Panchal: [sonalyogesh@yahoo.com](mailto:sonalyogesh@yahoo.com)

Trupti Joshi: drjoshitrupti@gmail.com

**Supplementary Table 1.** Characteristics of differential serum metabolites of Rural and Urban PCOS women.

| **Name of Metabolites** | **Parent ion (m/z)** | **Ion mode** | **Class of Compound** | **Fold Change (Rural/Urban)** | **log2(FC)** |
| --- | --- | --- | --- | --- | --- |
| DG (20:2n6/0:0/22:2n6) | 701.61 | Positive | Glycerol-based lipids | 17.874 | 4.1598 |
| DG (22:5(4Z,7Z,10Z,13Z,19Z)-O (16,17)/0:0/10:0) | 593.39 | Negative | Glycerol-based lipids | 1.3779 | 0.46243 |
| PA (18:1(9Z)-O (12,13) | 715.12 | Negative | Glycerophospholipid | 14.85 | 3.8924 |
| PA(PGD1/2:0) | 549.25 | Negative | Glycerophospholipid | 0.34251 | -1.5458 |
| PA (5-iso PGF2VI/18:3(9Z,12Z,15Z)) | 741.43 | Positive | Glycerophospholipid | 2.0458 | 1.0327 |
| PGP (18:1(9Z)-O (12,13) /i-12:0) | 785.38 | Negative | Glycerophospholipid | 1.5336 | 0.61695 |
| Palmitone | 451.47 | Positive | Unsaturated fatty acids | 4.7394 | 2.2447 |
| 3-hydroxyicosanoic Acid | 327.33 | Negative | Unsaturated fatty acids | 0.3646 | -1.4556 |
| Octadec-5-enoic acid | 283.26 | Positive | Unsaturated fatty acids | 0.57289 | -0.80366 |
| Glycerol tripropanoate | 281.1 | Negative | Triglycerides | 0.98859 | -0.01655 |
| O-(17-Carboxyheptadecanoyl) carnitine | 399.28 | Positive | Fatty acid metabolism | 1.9257 | 0.94538 |
| PS (24:0/24:0) | 960.8 | Positive | Phospholipid | 0.64655 | -0.62917 |
| LysoPC (14:0/0:0) | 488.31 | Negative | Phospholipid | 1.0961 | 0.13241 |
| Xanthosine 5-triphosphate | 524.98 | Positive | Nucleotide derivative | 8.8874 | 3.1518 |
| GDP-4-Dehydro-6-L-deoxygalactose | 586.72 | Negative | Sugar Nucleotides | 0.74847 | -0.41799 |
| UDP-beta-L-arabino furanose | 554.97 | Negative | Carbohydrate | 0.14642 | -2.7718 |
| Cer(d20:1/LTE4) | 749.56 | Positive | Ceramides | 5.0833 | 2.3458 |
| Cer(d18:1/22:0) | 642.06 | Negative | Ceramides | 4.8503 | 2.2781 |
| Cer(d18:0/12:0) | 482.81 | Negative | Ceramides | 0.98479 | -0.022114 |
| Cer(t18:0/20:3(8Z,11Z,14Z)-2OH (5,6)) | 638.53 | Positive | Ceramides | 2.337 | 1.2246 |
| 3-O-Sulfogalactosylceramide (d18:1/14:0) | 752.5 | Positive | Ceramides | 0.49472 | -1.0153 |
| 2-Methyloctacosane | 409.47 | Positive | Hydrocarbon | 4.7551 | 2.2495 |
| 14-Hentriacontanol | 453.46 | Positive | Long-chain alcohol | 0.33051 | -1.5972 |
| Triphosphate | 256.92 | Negative | Energy metabolism | 2.925 | 1.5484 |
| Adenosine tetraphosphate | 585.94 | Negative | Purine nucleotides | 0.39498 | -1.3401 |
| Heme | 614.17 | Negative | Porphyrins | 2.5184 | 1.3325 |
| N-Acetylsphinganine | 344.32 | Positive | Sphingolipids | 0.59602 | -0.74656 |
| Succinobucol | 615.29 | Negative | Antioxidants | 1.6755 | 0.74462 |
| 4-Ethyl-2-heptylthiazole | 212.17 | Positive | Thiazoles | 1.5505 | 0.63277 |
| Androstane-3,17-diol dipropionate | 405.3 | Positive | Androgen | 1.5336 | 0.61695 |
| Trichloroethanol glucuronide | 322.93 | Negative | Glucuronide Conjugates | 1.5336 | 0.61695 |
| ADP-ribose 1" -2" cyclic phosphate | 641.91 | Negative | Cyclic ADP-Ribose Phosphates | 1.3949 | 0.48018 |
| Stigmasteryl stearate | 679.67 | Positive | Sterol ester | 0.75764 | -0.40041 |
| Epifisetinidol-(4beta->8)-catechin | 583.11 | Negative | Polyphenols | 0.83147 | -0.26626 |
| 3b,6a-Dihydroxy-alpha-ionol 9-[apiosyl-(1->6)-glucoside] | 521.26 | Positive | O-acyl carbohydrate | 1.1415 | 0.1909 |
| 4-Hydroxy-17beta-estradiol-2-S-glutathione | 594.28 | Positive | Estrogen conjugates | 1.0549 | 0.077071 |
| Malathion dicarboxylic acid | 294.95 | Negative | Fatty Acyls | 1.0211 | 0.03009 |
