# Supplementary material for: Comparing the metabolomic landscape of polycystic ovary syndrome within urban and rural environments
Source: Commun Med (Lond). 2025 Jul 1;5:253. doi: 10.1038/s43856-025-00985-6 (PMC12214864; doi:10.1038/s43856-025-00985-6)
Supplement: Supplementary file 3 — Supplementary Data 2 [file 43856_2025_985_MOESM3_ESM.docx]

**Comparing the Metabolomic Landscape of Polycystic Ovary Syndrome within Urban and Rural Environments**

Jalpa Patel^1^, Hiral Chaudhary^1^, Abhishek Chudasama^1^, Jaydeep Panchal^2^, Akanksha Trivedi^2^, Sonal Panchal^3^, Trupti Joshi^4^, Rushikesh Joshi^1*^

^1^Department of Biochemistry and Forensic Science, University School of Sciences, Gujarat University, Ahmedabad-380009, Gujarat, India.

^2^Advait Theragnostics Pvt Ltd, Ahmedabad- 380009, Gujarat, India.

^3^Dr. Nagori's Institute for Infertility and IVF, Ahmedabad-380009, Gujarat, India.

^4^Urmi Hospital, Umreth-388220, Anand, Gujarat, India.

***Correspondence:**

Dr. Rushikesh Joshi, ​

Assistant Professor,

Department of Biochemistry & Forensic Science,

University School of Sciences,

Gujarat University, Ahmedabad-380009, India.

Email ID: [rushikeshjoshi@gujaratuniversity.ac.in](mailto:rushikeshjoshi@gujaratuniversity.ac.in)

**Author’s information**

Jalpa Patel: [jalpa.patel515@gmail.com](mailto:jalpa.patel515@gmail.com)

Hiral Chaudhary: [hiralchaudhary54@gmail.com](mailto:hiralchaudhary54@gmail.com)

Akanksha Trivedi: [akanksha.m1323@gmail.com](mailto:akanksha.m1323@gmail.com)

Abhishek Chudasama: [abhichudasama@gmail.com](mailto:abhichudasama@gmail.com)

Jaydeep Panchal: panchaljaydeep80@gmail.com

Sonal Panchal: [sonalyogesh@yahoo.com](mailto:sonalyogesh@yahoo.com)

Trupti Joshi: drjoshitrupti@gmail.com

**Supplementary Table 2.** Volcano plot analysis of differential metabolites.

| **Name of metabolites** | **Fold change (FC)** | **Log_2_(FC)** | **Raw. P value** | **-log10 p value** |
| --- | --- | --- | --- | --- |
| Palmitone | 4.7394 | 2.2447 | 4.40E-06 | 5.357 |
| UDP-beta-L-arabino furanose | 0.14642 | -2.7718 | 1.60E-05 | 4.7951 |
| 14-Hentriacontanol | 0.33051 | -1.5972 | 0.000209 | 3.6808 |
| Cer(d18:1/22:0) | 4.8503 | 2.2781 | 0.000219 | 3.6594 |
| 2-Methyloctacosane | 4.7551 | 2.2495 | 0.002839 | 2.5468 |
| Cer(d20:1/LTE4) | 5.0833 | 2.3458 | 0.004704 | 2.3276 |
| Adenosine tetraphosphate | 0.39498 | -1.3401 | 0.009377 | 2.0279 |
| Heme | 2.5184 | 1.3325 | 0.011993 | 1.9211 |
| PA (5-iso PGF2VI/18:3(9Z,12Z,15Z)) | 2.0458 | 1.0327 | 0.012155 | 1.9152 |
| PA (18:1(9Z)-O (12,13) | 14.85 | 3.8924 | 0.01466 | 1.8339 |
| 3-hydroxyicosanoic Acid | 0.3646 | -1.4556 | 0.016183 | 1.791 |
| Triphosphate | 2.925 | 1.5484 | 0.016781 | 1.7752 |
| Xanthosine 5-triphosphate  PA(PGD1/2:0) | 8.8874 | 3.1518 | 0.026723 | 1.5731 |
| PA(PGD1/2:0) | 0.34251 | -1.5458 | 0.032577 | 1.4871 |
| Cer(t18:0/20:3(8Z,11Z,14Z)-2OH (5,6)) | 2.337 | 1.2246 | 0.057469 | 1.2406 |
| DG (20:2n6/0:0/22:2n6) | 17.874 | 4.1598 | 0.062278 | 1.2057 |
