# Supplementary material for: Comparing the metabolomic landscape of polycystic ovary syndrome within urban and rural environments
Source: Commun Med (Lond). 2025 Jul 1;5:253. doi: 10.1038/s43856-025-00985-6 (PMC12214864; doi:10.1038/s43856-025-00985-6)
Supplement: Supplementary file 4 — Supplementary Data 3 [file 43856_2025_985_MOESM4_ESM.docx]

**Comparing the Metabolomic Landscape of Polycystic Ovary Syndrome within Urban and Rural Environments**

Jalpa Patel^1^, Hiral Chaudhary^1^, Abhishek Chudasama^1^, Jaydeep Panchal^2^, Akanksha Trivedi^2^, Sonal Panchal^3^, Trupti Joshi^4^, Rushikesh Joshi^1*^

^1^Department of Biochemistry and Forensic Science, University School of Sciences, Gujarat University, Ahmedabad-380009, Gujarat, India.

^2^Advait Theragnostics Pvt Ltd, Ahmedabad- 380009, Gujarat, India.

^3^Dr. Nagori's Institute for Infertility and IVF, Ahmedabad-380009, Gujarat, India.

^4^Urmi Hospital, Umreth-388220, Anand, Gujarat, India.

***Correspondence:**

Dr. Rushikesh Joshi, ​

Assistant Professor,

Department of Biochemistry & Forensic Science,

University School of Sciences,

Gujarat University, Ahmedabad-380009, India.

Email ID: [rushikeshjoshi@gujaratuniversity.ac.in](mailto:rushikeshjoshi@gujaratuniversity.ac.in)

**Author’s information**

Jalpa Patel: [jalpa.patel515@gmail.com](mailto:jalpa.patel515@gmail.com)

Hiral Chaudhary: [hiralchaudhary54@gmail.com](mailto:hiralchaudhary54@gmail.com)

Akanksha Trivedi: [akanksha.m1323@gmail.com](mailto:akanksha.m1323@gmail.com)

Abhishek Chudasama: [abhichudasama@gmail.com](mailto:abhichudasama@gmail.com)

Jaydeep Panchal: panchaljaydeep80@gmail.com

Sonal Panchal: [sonalyogesh@yahoo.com](mailto:sonalyogesh@yahoo.com)

Trupti Joshi: drjoshitrupti@gmail.com

**Supplementary Table 3.** Fold change analysis of differential metabolites.

| **Metabolites** | **Fold Change** | **log2(FC)** |
| --- | --- | --- |
| DG(20:2n6/0:0/22:2n6) | 17.874 | 4.1598 |
| PA(18:1(9Z)-O(12,13) | 14.85 | 3.8924 |
| Xanthosine 5-triphosphate | 8.8874 | 3.1518 |
| UDP-beta-L-arabinofuranose | 0.14642 | -2.7718 |
| Cer(d20:1/LTE4) | 5.0833 | 2.3458 |
| Cer(d18:1/22:0) | 4.8503 | 2.2781 |
| 2-Methyloctacosane | 4.7551 | 2.2495 |
| Palmitone | 4.7394 | 2.2447 |
| 14-Hentriacontanol | 0.33051 | -1.5972 |
| Triphosphate | 2.925 | 1.5484 |
| PA(PGD1/2:0) | 0.34251 | -1.5458 |
| 3-hydroxyicosanoic Acid | 0.3646 | -1.4556 |
| Adenosine tetraphosphate | 0.39498 | -1.3401 |
| Heme | 2.5184 | 1.3325 |
| Cer(t18:0/20:3(8Z,11Z,14Z)-2OH(5,6)) | 2.337 | 1.2246 |
| PA(5-iso PGF2VI/18:3(9Z,12Z,15Z)) | 2.0458 | 1.0327 |
| 3-O-Sulfogalactosylceramide (d18:1/14:0) | 0.49472 | -1.0153 |
