# Supplementary material for: Comparing the metabolomic landscape of polycystic ovary syndrome within urban and rural environments
Source: Commun Med (Lond). 2025 Jul 1;5:253. doi: 10.1038/s43856-025-00985-6 (PMC12214864; doi:10.1038/s43856-025-00985-6)
Supplement: Supplementary file 5 — Supplementary Data 4 [file 43856_2025_985_MOESM5_ESM.docx]

**Comparing the Metabolomic Landscape of Polycystic Ovary Syndrome within Urban and Rural Environments**

Jalpa Patel^1^, Hiral Chaudhary^1^, Abhishek Chudasama^1^, Jaydeep Panchal^2^, Akanksha Trivedi^2^, Sonal Panchal^3^, Trupti Joshi^4^, Rushikesh Joshi^1*^

^1^Department of Biochemistry and Forensic Science, University School of Sciences, Gujarat University, Ahmedabad-380009, Gujarat, India.

^2^Advait Theragnostics Pvt Ltd, Ahmedabad- 380009, Gujarat, India.

^3^Dr. Nagori's Institute for Infertility and IVF, Ahmedabad-380009, Gujarat, India.

^4^Urmi Hospital, Umreth-388220, Anand, Gujarat, India.

***Correspondence:**

Dr. Rushikesh Joshi, ​

Assistant Professor,

Department of Biochemistry & Forensic Science,

University School of Sciences,

Gujarat University, Ahmedabad-380009, India.

Email ID: [rushikeshjoshi@gujaratuniversity.ac.in](mailto:rushikeshjoshi@gujaratuniversity.ac.in)

**Author’s information**

Jalpa Patel: [jalpa.patel515@gmail.com](mailto:jalpa.patel515@gmail.com)

Hiral Chaudhary: [hiralchaudhary54@gmail.com](mailto:hiralchaudhary54@gmail.com)

Akanksha Trivedi: [akanksha.m1323@gmail.com](mailto:akanksha.m1323@gmail.com)

Abhishek Chudasama: [abhichudasama@gmail.com](mailto:abhichudasama@gmail.com)

Jaydeep Panchal: panchaljaydeep80@gmail.com

Sonal Panchal: [sonalyogesh@yahoo.com](mailto:sonalyogesh@yahoo.com)

Trupti Joshi: drjoshitrupti@gmail.com

**Supplementary Table 4.** T-test analysis of differential metabolites between Rural PCOS and Urban PCOS groups.

| **Name of metabolites** | **t.stat** | **p.value** | **p val -log10** | **FDR** |
| --- | --- | --- | --- | --- |
| Palmitone | 5.6757 | 4.40E-06 | 5.357 | 0.000163 |
| UDP-beta-L-arabinofuranose | -5.2005 | 1.60E-05 | 4.7951 | 0.000297 |
| 14-Hentriacontanol | -4.2599 | 0.000209 | 3.6808 | 0.002027 |
| Cer(d18:1/22:0) | 4.2417 | 0.000219 | 3.6594 | 0.002027 |
| 2-Methyloctacosane | 3.2716 | 0.002839 | 2.5468 | 0.02101 |
| Cer(d20:1/LTE4) | 3.0715 | 0.004704 | 2.3276 | 0.029005 |
| Adenosine tetraphosphate | -2.79 | 0.009377 | 2.0279 | 0.044975 |
| Stigmasteryl stearate | -2.7423 | 0.010513 | 1.9783 | 0.044975 |
| Heme | 2.687 | 0.011993 | 1.9211 | 0.044975 |
| PA(5-iso PGF2VI/18:3(9Z,12Z,15Z)) | 2.6813 | 0.012155 | 1.9152 | 0.044975 |
| PA(18:1(9Z)-O(12,13) | 2.6016 | 0.01466 | 1.8339 | 0.047762 |
| 3-hydroxyicosanoic Acid | -2.5592 | 0.016183 | 1.791 | 0.047762 |
| Triphosphate | 2.5435 | 0.016781 | 1.7752 | 0.047762 |
