# Supplementary material for: Comparing the metabolomic landscape of polycystic ovary syndrome within urban and rural environments
Source: Commun Med (Lond). 2025 Jul 1;5:253. doi: 10.1038/s43856-025-00985-6 (PMC12214864; doi:10.1038/s43856-025-00985-6)
Supplement: Supplementary file 6 — Supplementary Data 5 [file 43856_2025_985_MOESM6_ESM.docx]

**Comparing the Metabolomic Landscape of Polycystic Ovary Syndrome within Urban and Rural Environments**

Jalpa Patel^1^, Hiral Chaudhary^1^, Abhishek Chudasama^1^, Jaydeep Panchal^2^, Akanksha Trivedi^2^, Sonal Panchal^3^, Trupti Joshi^4^, Rushikesh Joshi^1*^

^1^Department of Biochemistry and Forensic Science, University School of Sciences, Gujarat University, Ahmedabad-380009, Gujarat, India.

^2^Advait Theragnostics Pvt Ltd, Ahmedabad- 380009, Gujarat, India.

^3^Dr. Nagori's Institute for Infertility and IVF, Ahmedabad-380009, Gujarat, India.

^4^Urmi Hospital, Umreth-388220, Anand, Gujarat, India.

***Correspondence:**

Dr. Rushikesh Joshi, ​

Assistant Professor,

Department of Biochemistry & Forensic Science,

University School of Sciences,

Gujarat University, Ahmedabad-380009, India.

Email ID: [rushikeshjoshi@gujaratuniversity.ac.in](mailto:rushikeshjoshi@gujaratuniversity.ac.in)

**Author’s information**

Jalpa Patel: [jalpa.patel515@gmail.com](mailto:jalpa.patel515@gmail.com)

Hiral Chaudhary: [hiralchaudhary54@gmail.com](mailto:hiralchaudhary54@gmail.com)

Akanksha Trivedi: [akanksha.m1323@gmail.com](mailto:akanksha.m1323@gmail.com)

Abhishek Chudasama: [abhichudasama@gmail.com](mailto:abhichudasama@gmail.com)

Jaydeep Panchal: panchaljaydeep80@gmail.com

Sonal Panchal: [sonalyogesh@yahoo.com](mailto:sonalyogesh@yahoo.com)

Trupti Joshi: drjoshitrupti@gmail.com

**Supplementary Table 5.** The rank of the metabolites by their contribution to the group differentiation with scores by Partial Least Squares Discriminant Analysis.

| **Name of Metabolites** | **V1** | **V2** |
| --- | --- | --- |
| Palmitone | 1.887804 | 0.799193 |
| UDP-beta-L-arabinofuranose | 1.859169 | 0.460653 |
| 14-Hentriacontanol | 1.719961 | 0.599969 |
| Cer(d18:1/22:0) | 1.66294 | 0.950591 |
| 2-Methyloctacosane | 1.456776 | 1.002091 |
| Cer(d20:1/LTE4) | 1.41953 | 0.657988 |
| Heme | 1.22604 | 0.932414 |
| Adenosine tetraphosphate | 1.19976 | 0.514963 |
| PA (5-iso PGF2VI/18:3(9Z,12Z,15Z)) | 1.195796 | 1.214488 |
| PA (18:1(9Z)-O (12,13) | 1.194791 | 0.964525 |
| Stigmasteryl stearate | 1.150015 | 1.003923 |
| Triphosphate | 1.145283 | 0.822725 |
| 3-hydroxyicosanoic Acid | 1.122842 | 0.480283 |
| Xanthosine 5-triphosphate | 1.081437 | 0.766777 |
| PA(PGD1/2:0) | 1.074629 | 0.703222 |
| Cer(t18:0/20:3(8Z,11Z,14Z)-2OH (5,6)) | 1.021964 | 1.25222 |
| DG (22:5(4Z,7Z,10Z,13Z,19Z)-O (16,17)/0:0/10:0) | 1.013569 | 1.048559 |
| DG (20:2n6/0:0/22:2n6) | 0.971896 | 0.847899 |
| Succinobucol | 0.863747 | 1.282533 |
| Androstane-3,17-diol dipropionate | 0.846519 | 1.240674 |
| PGP (18:1(9Z)-O (12,13) /i-12:0) | 0.846429 | 1.240547 |
| Trichloroethanol glucuronide | 0.846412 | 1.240523 |
| O-(17-Carboxyheptadecanoyl) carnitine | 0.754068 | 1.034796 |
| 3-O-Sulfogalactosylceramide (d18:1/14:0) | 0.641197 | 0.85735 |
| 4-Ethyl-2-heptylthiazole | 0.532005 | 0.307395 |
| ADP-ribose 1""-2"" cyclic phosphate | 0.448174 | 1.50018 |
| Octadec-5-enoic acid | 0.43317 | 1.073105 |
| N-Acetylsphinganine | 0.416561 | 1.087885 |
| PS (24:0/24:0) | 0.279874 | 1.049515 |
| GDP-4-Dehydro-6-L-deoxygalactose | 0.26172 | 1.029401 |
| Malathion dicarboxylic acid | 0.244012 | 1.347869 |
| Glycerol tripropanoate | 0.237094 | 1.204371 |
| Epifisetinidol-(4beta->8)-catechin | 0.200825 | 0.716688 |
| Cer(d18:0/12:0) | 0.153248 | 1.080585 |
| LysoPC (14:0/0:0) | 0.149375 | 0.639452 |
| 4-Hydroxy-17beta-estradiol-2-S-glutathione | 0.101844 | 1.417564 |
| 3b,6a-Dihydroxy-alpha-ionol 9-[apiosyl-(1->6)-glucoside] | 0.060959 | 1.117189 |
