# Supplementary material for: Comparing the metabolomic landscape of polycystic ovary syndrome within urban and rural environments
Source: Commun Med (Lond). 2025 Jul 1;5:253. doi: 10.1038/s43856-025-00985-6 (PMC12214864; doi:10.1038/s43856-025-00985-6)
Supplement: Supplementary file 7 — Supplementary Data 6 [file 43856_2025_985_MOESM7_ESM.docx]

**Comparing the Metabolomic Landscape of Polycystic Ovary Syndrome within Urban and Rural Environments**

Jalpa Patel^1^, Hiral Chaudhary^1^, Abhishek Chudasama^1^, Jaydeep Panchal^2^, Akanksha Trivedi^2^, Sonal Panchal^3^, Trupti Joshi^4^, Rushikesh Joshi^1*^

^1^Department of Biochemistry and Forensic Science, University School of Sciences, Gujarat University, Ahmedabad-380009, Gujarat, India.

^2^Advait Theragnostics Pvt Ltd, Ahmedabad- 380009, Gujarat, India.

^3^Dr. Nagori's Institute for Infertility and IVF, Ahmedabad-380009, Gujarat, India.

^4^Urmi Hospital, Umreth-388220, Anand, Gujarat, India.

***Correspondence:**

Dr. Rushikesh Joshi, ​

Assistant Professor,

Department of Biochemistry & Forensic Science,

University School of Sciences,

Gujarat University, Ahmedabad-380009, India.

Email ID: [rushikeshjoshi@gujaratuniversity.ac.in](mailto:rushikeshjoshi@gujaratuniversity.ac.in)

**Author’s information**

Jalpa Patel: [jalpa.patel515@gmail.com](mailto:jalpa.patel515@gmail.com)

Hiral Chaudhary: [hiralchaudhary54@gmail.com](mailto:hiralchaudhary54@gmail.com)

Akanksha Trivedi: [akanksha.m1323@gmail.com](mailto:akanksha.m1323@gmail.com)

Abhishek Chudasama: [abhichudasama@gmail.com](mailto:abhichudasama@gmail.com)

Jaydeep Panchal: panchaljaydeep80@gmail.com

Sonal Panchal: [sonalyogesh@yahoo.com](mailto:sonalyogesh@yahoo.com)

Trupti Joshi: [drjoshitrupti@gmail.com](mailto:drjoshitrupti@gmail.com)

**Supplementary Table 6.** Significant differential expression of metabolites by Significant analysis of microarrays (SAM)

| **Name of Metabolites** | **d.value** | **stdev** | **rawp** | **q.value** |
| --- | --- | --- | --- | --- |
| Palmitone | -2.30 | 0.25 | 0.00 | 0.00 |
| UDP-beta-L-arabinofuranose | 2.17 | 0.27 | 0.00 | 0.00 |
| 14-Hentriacontanol | 1.87 | 0.29 | 0.00 | 0.00 |
| Cer(d18:1/22:0) | -1.86 | 0.29 | 0.00 | 0.00 |
| 2-Methyloctacosane | -1.50 | 0.32 | 0.00 | 0.00 |
| Cer(d20:1/LTE4) | -1.42 | 0.32 | 0.00 | 0.00 |
| Adenosine tetraphosphate | 1.31 | 0.33 | 0.01 | 0.00 |
| Stigmasteryl stearate | 1.29 | 0.33 | 0.01 | 0.00 |
| Heme | -1.27 | 0.33 | 0.01 | 0.00 |
| PA(5-iso PGF2VI/18:3(9Z,12Z,15Z)) | -1.26 | 0.33 | 0.01 | 0.00 |
| PA(18:1(9Z)-O(12,13) | -1.23 | 0.33 | 0.01 | 0.00 |
| 3-hydroxyicosanoic Acid | 1.21 | 0.34 | 0.01 | 0.00 |
| Triphosphate | -1.21 | 0.34 | 0.01 | 0.00 |
| Xanthosine 5-triphosphate | -1.12 | 0.34 | 0.03 | 0.01 |
| DG(22:5(4Z,7Z,10Z,13Z,19Z)-O(16,17)/0:0/10:0) | -1.12 | 0.34 | 0.03 | 0.01 |
| PA(PGD1/2:0) | 1.08 | 0.34 | 0.03 | 0.01 |
| Cer(t18:0/20:3(8Z,11Z,14Z)-2OH(5,6)) | -0.96 | 0.35 | 0.06 | 0.02 |
| DG(20:2n6/0:0/22:2n6) | -0.94 | 0.35 | 0.07 | 0.02 |
| Androstane-3,17-diol dipropionate | -0.94 | 0.35 | 0.07 | 0.02 |
| PGP(18:1(9Z)-O(12,13) /i-12:0) | -0.94 | 0.35 | 0.07 | 0.02 |
| Trichloroethanol glucuronide | -0.94 | 0.35 | 0.07 | 0.02 |
| Succinobucol | -0.84 | 0.35 | 0.10 | 0.02 |
| 3-O-Sulfogalactosylceramide (d18:1/14:0) | 0.70 | 0.36 | 0.18 | 0.04 |
| O-(17-Carboxyheptadecanoyl)carnitine | -0.70 | 0.36 | 0.18 | 0.04 |
| 4-Ethyl-2-heptylthiazole | -0.55 | 0.36 | 0.30 | 0.06 |
