# Supplementary material for: Comparing the metabolomic landscape of polycystic ovary syndrome within urban and rural environments
Source: Commun Med (Lond). 2025 Jul 1;5:253. doi: 10.1038/s43856-025-00985-6 (PMC12214864; doi:10.1038/s43856-025-00985-6)
Supplement: Supplementary file 8 — Supplementary Data 7 [file 43856_2025_985_MOESM8_ESM.docx]

**Comparing the Metabolomic Landscape of Polycystic Ovary Syndrome within Urban and Rural Environments**

Jalpa Patel^1^, Hiral Chaudhary^1^, Abhishek Chudasama^1^, Jaydeep Panchal^2^, Akanksha Trivedi^2^, Sonal Panchal^3^, Trupti Joshi^4^, Rushikesh Joshi^1*^

^1^Department of Biochemistry and Forensic Science, University School of Sciences, Gujarat University, Ahmedabad-380009, Gujarat, India.

^2^Advait Theragnostics Pvt Ltd, Ahmedabad- 380009, Gujarat, India.

^3^Dr. Nagori's Institute for Infertility and IVF, Ahmedabad-380009, Gujarat, India.

^4^Urmi Hospital, Umreth-388220, Anand, Gujarat, India.

***Correspondence:**

Dr. Rushikesh Joshi, ​

Assistant Professor,

Department of Biochemistry & Forensic Science,

University School of Sciences,

Gujarat University, Ahmedabad-380009, India.

Email ID: [rushikeshjoshi@gujaratuniversity.ac.in](mailto:rushikeshjoshi@gujaratuniversity.ac.in)

**Author’s information**

Jalpa Patel: [jalpa.patel515@gmail.com](mailto:jalpa.patel515@gmail.com)

Hiral Chaudhary: [hiralchaudhary54@gmail.com](mailto:hiralchaudhary54@gmail.com)

Akanksha Trivedi: [akanksha.m1323@gmail.com](mailto:akanksha.m1323@gmail.com)

Abhishek Chudasama: [abhichudasama@gmail.com](mailto:abhichudasama@gmail.com)

Jaydeep Panchal: panchaljaydeep80@gmail.com

Sonal Panchal: [sonalyogesh@yahoo.com](mailto:sonalyogesh@yahoo.com)

Trupti Joshi: [drjoshitrupti@gmail.com](mailto:drjoshitrupti@gmail.com)

**Supplementary Table 7** Receiver Operating Characteristic (ROC) analysis for Biomarker identification.

| **Metabolites** | **AUC** | **T-tests** | **log2 FC** |
| --- | --- | --- | --- |
| Palmitone | 0.9107 | 0.000043 | 2.0404 |
| 14-Hentriacontanol | 0.8526 | 0.00020 | -1.614 |
| Cer (d18:1/ 22:0) | 0.8482 | 0.00021 | 2.030 |
| UDP-beta-L-arabino furanose | 0.8437 | 0.000016 | -3.001 |
| DG (20:2n6/0:0/22:2n6) | 0.8125 | 0.0622 | 4.7088 |
| PA (5-iso PGF2VI/18:3 (9Z,12Z,15Z) | 0.8125 | 0.0121 | 1.0616 |
| Xanthosine 5-triphosphate | 0.7767 | 0.0267 | 3.1872 |
| Heme | 0.7767 | 0.0119 | 1.1819 |
| Triphosphate | 0.7723 | 0.0167 | 1.3099 |
| Cer (d20:1/ LTE-4) | 0.7678 | 0.0047 | 2.0199 |
| DG (22:5 (4Z, 7Z,10Z, 19Z)-O (16,17) /0:0/ 10:0) | 0.7678 | 0.0270 | 0.3427 |
| 2-methyloctacosane | 0.7410 | 0.0028 | 1.8211 |
| Adenosine tetraphosphate | 0.7276 | 0.0093 | -1.4174 |
| PA (18:1 (9Z)-O (12,13) | 0.7232 | 0.0146 | 3.2091 |
| Stigmasteryl stearate | 0.7187 | 0.0105 | -0.6522 |
| O-(17-carboxyheptadecanoyl) carnitine | 0.7053 | 0.1652 | 0.5830 |
| Androstane-3, 17diol dipropionate | 0.7053 | 0.0636 | 0.3625 |
| Cer (t18:0/ 20:3 (8Z, 11Z, 14Z)-2OH (5, 6) | 0.7053 | 0.0574 | 0.8125 |
| Tri chloroethanol glucuronide | 0.7053 | 0.0636 | 0.3625 |
| PGP (18:1 (9Z)-O (12, 13)/ i-12:0) | 0.7053 | 0.0636 | 0.3625 |
| Succinobucol | 0.6919 | 0.0943 | 0.3625 |
| ADP-ribose 1’’-2’’ cyclic phosphate | 0.6875 | 0.4219 | 0.2851 |
| 3-hydroxyicosanoic acid | 0.6473 | 0.0161 | -1.8217 |
| 4-Ethyl-2-heptylthiazole | 0.6383 | 0.2771 | 0.2533 |
| Glycerol triphosphate | 0.6339 | 0.6129 | -0.3219 |
| PA (PGD1/ 2:0) | 0.6339 | 0.0325 | -1.9657 |
| Malathion dicarboxylic acid | 0.6294 | 0.5769 | -0.3219 |
| Cer (d18:0/12:0) | 0.625 | 0.7573 | -0.3032 |
