# Supplementary material for: Comparing the metabolomic landscape of polycystic ovary syndrome within urban and rural environments
Source: Commun Med (Lond). 2025 Jul 1;5:253. doi: 10.1038/s43856-025-00985-6 (PMC12214864; doi:10.1038/s43856-025-00985-6)
Supplement: Supplementary file 9 — Supplementary Data 8 [file 43856_2025_985_MOESM9_ESM.docx]

**Comparing the Metabolomic Landscape of Polycystic Ovary Syndrome within Urban and Rural Environments**

Jalpa Patel^1^, Hiral Chaudhary^1^, Abhishek Chudasama^1^, Jaydeep Panchal^2^, Akanksha Trivedi^2^, Sonal Panchal^3^, Trupti Joshi^4^, Rushikesh Joshi^1*^

^1^Department of Biochemistry and Forensic Science, University School of Sciences, Gujarat University, Ahmedabad-380009, Gujarat, India.

^2^Advait Theragnostics Pvt Ltd, Ahmedabad- 380009, Gujarat, India.

^3^Dr. Nagori's Institute for Infertility and IVF, Ahmedabad-380009, Gujarat, India.

^4^Urmi Hospital, Umreth-388220, Anand, Gujarat, India.

***Correspondence:**

Dr. Rushikesh Joshi, ​

Assistant Professor,

Department of Biochemistry & Forensic Science,

University School of Sciences,

Gujarat University, Ahmedabad-380009, India.

Email ID: [rushikeshjoshi@gujaratuniversity.ac.in](mailto:rushikeshjoshi@gujaratuniversity.ac.in)

**Author’s information**

Jalpa Patel: [jalpa.patel515@gmail.com](mailto:jalpa.patel515@gmail.com)

Hiral Chaudhary: [hiralchaudhary54@gmail.com](mailto:hiralchaudhary54@gmail.com)

Akanksha Trivedi: [akanksha.m1323@gmail.com](mailto:akanksha.m1323@gmail.com)

Abhishek Chudasama: [abhichudasama@gmail.com](mailto:abhichudasama@gmail.com)

Jaydeep Panchal: panchaljaydeep80@gmail.com

Sonal Panchal: [sonalyogesh@yahoo.com](mailto:sonalyogesh@yahoo.com)

Trupti Joshi: [drjoshitrupti@gmail.com](mailto:drjoshitrupti@gmail.com)

**Supplementary Table 8** Pathway enrichment analysis of differential metabolites between PCOS and control groups.

| **Name of Pathway** | **Total Compound** | **Hits** | **Raw p** | **log₁₀(p)** | **Holm adjust** | **False discovery rate** | **Impact** |
| --- | --- | --- | --- | --- | --- | --- | --- |
| Sphingolipid metabolism | 32 | 3 | 0.00 | 2.93 | 0.01 | 0.01 | 0.31 |
| Porphyrin metabolism | 31 | 1 | 0.01 | 1.92 | 0.06 | 0.04 | 0.16 |
| Metabolism of xenobiotics by cytochrome P450 | 68 | 1 | 0.06 | 1.20 | 0.19 | 0.10 | 0.00 |
| Biosynthesis of unsaturated fatty acids | 36 | 1 | 0.41 | 0.39 | 0.81 | 0.49 | 0.00 |
| Glycerophospholipid metabolism | 36 | 1 | 0.85 | 0.07 | 0.85 | 0.85 | 0.02 |
